# Decoding Cancer Variants of Unknown Significance for Helicase–Nuclease–RPA Complexes Orchestrating DNA Repair During Transcription and Replication

**DOI:** 10.3389/fmolb.2021.791792

**Published:** 2021-12-14

**Authors:** Susan E. Tsutakawa, Albino Bacolla, Panagiotis Katsonis, Amer Bralić, Samir M. Hamdan, Olivier Lichtarge, John A. Tainer, Chi-Lin Tsai

**Affiliations:** ^1^ Molecular Biophysics and Integrated Bioimaging, Lawrence Berkeley National Laboratory, Berkeley, CA, United States; ^2^ Department of Molecular and Cellular Oncology, University of Texas M.D. Anderson Cancer Center, Houston, TX, United States; ^3^ Department of Molecular and Human Genetics, Baylor College of Medicine, Houston, TX, United States; ^4^ Laboratory of DNA Replication and Recombination, Biological and Environmental Sciences and Engineering Division, King Abdullah University of Science and Technology (KAUST), Thuwal, Saudi Arabia; ^5^ Department of Cancer Biology, University of Texas M.D. Anderson Cancer Center, Houston, TX, United States

**Keywords:** protein structure, evolutionary action, VUS, cancer mutations, helicase-nuclease, transcription, nucleotide excision repair, replication forks

## Abstract

All tumors have DNA mutations, and a predictive understanding of those mutations could inform clinical treatments. However, 40% of the mutations are variants of unknown significance (VUS), with the challenge being to objectively predict whether a VUS is pathogenic and supports the tumor or whether it is benign. To objectively decode VUS, we mapped cancer sequence data and evolutionary trace (ET) scores onto crystallography and cryo-electron microscopy structures with variant impacts quantitated by evolutionary action (EA) measures. As tumors depend on helicases and nucleases to deal with transcription/replication stress, we targeted helicase–nuclease–RPA complexes: (1) XPB-XPD (within TFIIH), XPF-ERCC1, XPG, and RPA for transcription and nucleotide excision repair pathways and (2) BLM, EXO5, and RPA plus DNA2 for stalled replication fork restart. As validation, EA scoring predicts severe effects for most disease mutations, but disease mutants with low ET scores not only are likely destabilizing but also disrupt sophisticated allosteric mechanisms. For sites of disease mutations and VUS predicted to be severe, we found strong co-localization to ordered regions. Rare discrepancies highlighted the different survival requirements between disease and tumor mutations, as well as the value of examining proteins within complexes. In a genome-wide analysis of 33 cancer types, we found correlation between the number of mutations in each tumor and which pathways or functional processes in which the mutations occur, revealing different mutagenic routes to tumorigenesis. We also found upregulation of ancient genes including BLM, which supports a non-random and concerted cancer process: reversion to a unicellular, proliferation-uncontrolled, status by breaking multicellular constraints on cell division. Together, these genes and global analyses challenge the binary “driver” and “passenger” mutation paradigm, support a gradient impact as revealed by EA scoring from moderate to severe at a single gene level, and indicate reduced regulation as well as activity. The objective quantitative assessment of VUS scoring and gene overexpression in the context of functional interactions and pathways provides insights for biology, oncology, and precision medicine.

## Introduction

Tumor mutational burden as well as increasing findings of somatic and germline variants from next-generation sequencing (NGS) feed the need to combine molecular and mechanistic knowledge (Chan et al., 2019; Cobain et al., 2021). NGS strikingly reveals that ∼40% of the total inherited variants are variants of unknown significance (VUS), which are mostly missense substitutions or in-frame insertions/deletions (Balmana et al., 2016; Federici and Soddu, 2020). Because there is limited knowledge of the impacts of VUS on protein structures and functions (MacArthur et al., 2014), these VUS cannot be applied to “adaptive design” treatments based on biomarker profiling to improve outcomes and that are replacing “tumor type-centered” treatments (Tsimberidou et al., 2020). Unlike insertion/deletion/frameshifts that are most likely to result in truncated and misfolded proteins that are degraded, missense substitutions are more challenging to interpret as their impact is less evident. The apparently random distribution of VUS further increases the difficulties of analyzing missense mutations. Even in high-risk cancer susceptibility genes, the determination of probable pathogenicity or clinical impact of VUS remains a monumental task.

An objective and quantitative means to decode VUS in order to identify important and impactful variants is critical to provide specific testable hypotheses and assist precision molecular medicine and mechanistic clinical applications in the absence of experimental data. Ideally, computational tools will enable clinical studies to better target dynamic changes in tumor abnormalities, reduce residual disease, and eradicate subclones that confer resistance to treatment (Tsimberidou et al., 2020). Highly accurate structure prediction algorithms such as AlphaFold or RoseTTAFold enable variant mapping onto three-dimensional structures for evaluating their structural and functional impacts (Kryshtafovych et al., 2019; Baek et al., 2021; Jumper et al., 2021). However, an objective and quantitative means to select the most severe variants over millions of somatic and germline variants remains a challenge.

Here, we developed, tested, and applied an approach to combine atomic protein structures with evolutionary trace (ET) and evolutionary action (EA) computational analyses (Lichtarge et al., 1996; Katsonis and Lichtarge, 2014). These analyses efficiently identified and objectively assessed the most impactful germline and somatic variants in helicase–nuclease–RPA complexes, which are vital for resolving transcription and replication stress that are common features of cancer cells (Gomez-Gonzalez and Aguilera, 2019; Ubhi and Brown, 2019; Bowry et al., 2021). Going beyond sequence conservation, ET considers sequence changes that occur and that are retained through evolution to providing those organisms with a functional advantage. ET scores the functional sensitivity of each sequence position to an amino acid residue substitution based on the observed divergence among evolutionary-related sequences, and it ranks each residue from 0 (most important) to 100 (least important). EA further represents the evolutionary fitness effect of a single missense mutation. It ranks onto a spectrum of genotype perturbation from 100 (pathogenic or loss of function) to 0 (benign or wild-type) based on the magnitude of the amino acid variation and the functional sensitivity of the site where the substitution occurs given by the ET score. Structures place ET and EA values into their three-dimensional context. Co-localized ET scores reliably identify active sites and allosteric pathway residues that have been tested experimentally (Yao et al., 2003; Rodriguez et al., 2010; Adikesavan et al., 2011; Wilkins et al., 2012) and inform molecular mechanisms of disease-causing missense mutations (Stefl et al., 2013). For example, ET scoring of DNA-PK identified the kinase active site as well as a seemingly randomly placed helix (Lees-Miller et al., 2021), which was later implicated in double-strand break (DSB)-induced dimerization to join DNA ends (Chen et al., 2021). EA is an untrained method making unbiased predictions, reflecting protein evolutionary fitness effect (Katsonis and Lichtarge, 2019). So, considering variants with EA score of 80 (EA80), the expectation is that about 80% of them will be found to be deleterious by an experimental assay or clinical association. We therefore applied combined ET/EA analyses that correlate with inherited disease manifestations in the context of specific cancer proteins and complexes of interest. In this way, we aim to provide a path to producing robust predictive information to test and potentially reassign many VUS into predicted (1) severe, (2) mix, and (3) benign tiers of clinical significance.

At the interface of repair with transcription and replication, helicase–nuclease–RPA partnerships have major roles in genome maintenance by directional unwinding and processing structured nucleic acids including double-stranded DNA (dsDNA) and alternate DNA structures. Their biological importance is underscored by cancers and genetic disorders that are linked to defects in their structures, assemblies, and activities. Yet, a robust experimental assay to classify sequence variants in these complexes is made unlikely by the observation that these dynamic assemblies are typically multi-functional. For example, tight regulation of helicase and nuclease activities enable their context-dependent role and restricts indiscriminate dsDNA unwinding and incision, which would otherwise promote genome instability. Part of this regulation emerges from direct helicase–nuclease–RPA interactions for complexes acting in both transcription and replication.

During transcription stress, collisions with replication forks can promote genome instability (Gomez-Gonzalez and Aguilera, 2019). In particular, the nascent RNA strand can base pair with its template DNA, displacing single-stranded DNA (ssDNA) from the non-template strand to form an R-loop structure. If not removed by helicases and nucleases, such R-loops can become blocks to transcription and replication that trigger DNA repair by the transcription-coupled nucleotide excision repair (TC-NER) nucleases XPG and XPF (Crossley et al., 2019). Indeed, TC-NER proteins may have first evolved from evolutionary pressure to resolve arrested transcription blocks to DNA replication (Sarker et al., 2005; Tsutakawa et al., 2020b). For TC-NER and NER in general, bulky DNA lesions that block transcription are removed as a 25–27 oligonucleotide (Staresincic et al., 2009; Hu et al., 2015; Li et al., 2018; Tsutakawa et al., 2020b). Within the multi-subunit Transcription Factor IIH (TFIIH), XPB and XPD are, respectively, ds and ssDNA translocases that work together as helicases to unwind the dsDNA around the lesion facilitated by XPA, with RPA binding the just-unpaired undamaged ssDNA strand (Kokic et al., 2019). XPG is the first nuclease recruited to the damage site by TFIIH and XPA, although, in solution, XPG first plays a structural role in the stabilization of a pre-incision complex. Subsequently, XPF–ERCC1 nuclease is recruited by XPA to incise the damaged strand 5′ to the lesion, forming the 3′ OH that initiates DNA synthesis and potentially triggers catalytic activation of XPG, which incises 3′ to the lesion (Staresincic et al., 2009; Orelli et al., 2010). Designing experimental assays to test variant function is complicated by the fact that these proteins play additional roles in the cell. For example, TFIIH (XPB and XPD) has an essential role in initiating transcription by opening dsDNA for RNA polymerase assembly (Rimel and Taatjes, 2018; Tsutakawa et al., 2020b). XPF-ERCC1 is required for cross-link DNA repair, DSB repair, telomere maintenance, and possibly repair of oxidative lesions (Kuraoka et al., 2000; Zhu et al., 2003; Kirschner et al., 2007; Ahmad et al., 2008). XPG plays roles with WRN helicase, which has strand annealing and editing roles at replication forks (Perry et al., 2006; Trego et al., 2011), in DNA base excision repair (BER) and in homology-directed repair (HDR) (Trego et al., 2016). RPA functions in DNA replication, mismatch repair (MMR), BER, and HDR (Caldwell and Spies, 2020).

During replication stress, stalled replication forks are processed by helicases and nucleases for repair and restart, and RPA binds ssDNA to recruit ATR kinase for regulation of stress response by activating fork remodelers, protectors, and restart complexes to ensure genome integrity (Saldivar et al., 2017). Stalled forks can be reversed by fork remodelers (SMARCAL1, HLTF, and ZRANB3), stabilized by fork protection factors (BRCA1/2, PARP1, and Fanconi anemia proteins), and restarted by helicase–nuclease complexes (BLM-EXO5, WRN-DNA2) to maintain genome stability (Thangavel et al., 2015; Liao et al., 2018; Hambarde et al., 2021). To prevent unscheduled nuclease degradation, efficient stalled replication fork restart by BLM-EXO5-RPA complex is a critical step to resume replication. Defects in BLM-EXO5 complex decrease replication fork restart frequency and result in increased frequencies of chromosomal radials and sister-chromatid exchange, characteristics of Bloom syndrome (BS) patients, who are susceptible to cancer (Cunniff et al., 2017; Hambarde et al., 2021). Furthermore, BLM-deficient cells increase genetic exchanges between homologous chromosomes, resulting in loss of heterozygosity (LOH), which typically increases in cancer and aging cells. Thus, understanding which missense mutations cause BLM-EXO5 defects is both exemplary and critical to predict and control the outcomes of cancer and other progressive diseases.

Based on the observations noted above, we reasoned that sequence variants that damage folding, activity, and context-dependent roles of helicase–nuclease–RPA complexes are likely to promote instability and oncogenesis. We therefore applied ET/EA analyses to these two prototypic helicase–nuclease–RPA systems: (1) XPB, XPD, XPF, XPG, and RPA for bulky lesion and cross-link repair through TC-NER and NER pathways; and (2) BLM, EXO5, and RPA plus DNA2 for stalled replication fork restart. These systems directly impact genomic stability that is lost in cancer and/or act in resistance against chemotherapy. Furthermore, the existence of single-site mutations with known disease phenotypes and known structures provide benchmarks to assess the accuracy of VUS scoring by EA. Layering ET (significance for function), autosomal recessive disease mutations (validation of EA scoring), and EA (tumor mutations with ranking of functional loss) onto available structures or structure predictions, we found new insights into protein mechanisms and predicted which VUS are most likely to negatively impact a protein’s function. Unexpectedly, there is a remarkable absence of negative selection in cancer on coding point mutations in essential functional regions (Martincorena et al., 2017), implying that cancer evolution has survival criteria distinct from those governing disease mutations. To complement EA/ET analyses, we created a genome-wide mutability map that reveals low mutability in conserved ancient genes such as *BLM* that are often overexpressed in cancer cells leading towards reverse evolution from the multicellular back to a primordial unicellular hyperproliferative state (Chen et al., 2015). The map also conversely supports our EA score assessment showing that *ERCC2* is selectively targeted for mutations in urinary tract cancers. The collective results inform catalytic and structural roles of multi-functional complexes by harnessing cancer mutations to map key sites for functional conformations, ATPase regulation, plus both protein and DNA interactions.

## Materials and Methods

### Bioinformatic Resources

Kaplan–Meier survival curves for patients with and without *ERCC5* mutations were obtained from cBioPortal (https://www.cbioportal.org) by selecting the “Curated set of non-redundant studies.” For Kaplan–Meier survival curves and the analyses of gene expression data in TCGA patients, we used an in-house pipeline, as reported (Hambarde et al., 2021). Scripts are available at https://github.com/abacolla. Signature mutations were obtained and analyzed as reported (Eckelmann et al., 2020).

### Source of Gene Mutations

Germline mutations reported to be causative of inherited disease were obtained from the Human Gene Mutation Database (HGMD^®^ Professional 2019.2) through an institutional license. In most cases, we selected the “DM” (disease mutation) classification, except in BLM, where we also kept “FP” (functional polymorphism) as validated variants with the loss of function but no known disease association yet (Stenson et al., 2014). Some disease mutations for ERCC4 (XPF) and DNA2 had inconsistency in residue numbers based on literatures and were corrected in HGMD (Matsumura et al., 1998; Ronchi et al., 2013). Somatic mutations in cancer genomes were obtained from the Catalogue Of Somatic Mutations In Cancer (COSMIC v92) at https://cancer.sanger.ac.uk/cosmic, file CosmicMutantExport.tsv. Germline mutations in BLM that were classified as VUS in ClinVar Miner database (https://clinvarminer.genetics.utah.edu/) were also used for EA analysis (Henrie et al., 2018).

### Evolutionary Trace Analysis

We performed ET analyses (Lichtarge et al., 1996; Mihalek et al., 2004) using the following query sequences: NP_000391 for ERCC2 (XPD), NP_000113 for ERCC3 (XPB), NP_005227 for ERCC4 (XPF), NP_000114 for ERCC5 (XPG), NP_000048 for BLM, NP_073611 for EXO5, NP_001073918 for DNA2, NP_002936 for RPA1, NP_002937 for RPA2, and NP_002938 for RPA3. We obtained homologous sequences for each query by BLAST searches using blastall 2.2.15 (Altschul et al., 1997) against the protein sequence databases NCBI nr, UniRef90, and Uniref100 (Pruitt et al., 2007; Suzek et al., 2015). We used a custom script to select homologous sequences for each query that represent different phylogenetic distances with the fewest possible alignment gaps. Then, we aligned the sequences using MUSCLE (Edgar, 2004) and run the ET analysis with the option “*position-specific gap-reducing real-valued trace*.” Although it is beneficial to use PyMOL 2.3.4 and the PyETV plugin (Lua and Lichtarge, 2010) to color-map the ET scores (red means most important and green means least important) on the PDB structures (see *Structural Modeling* section), we found that mapping the lowest scoring ET (most important) residues to structures provide an alternative viewpoint that is insightful. All genes (*ERCC2-3-4-5, BLM, EXO5, DNA2,* and *RPA*) with ET scores are listed in Supplementary Table S11. The first two tiers of low ET scored residues, e.g., ET < 2 (1st tier) and ET 2–4 (2nd tier), were mapped in the structures to have focused analyses, meaning too many mapped residues could obscure the most important sites. For example, for BLM, 1st-tier ET already has 59 residues scores as 4.96; thus, we only chose ET scores between 4.96 and 6.91 as 2nd tier to include extra 37 residues instead of ET ∼10 and analyze them separately. We found that this strategy can provide a cleaner map to identify important functional sites. The validation of ET analysis based on the known functional sites and identification of new functional site with experimental validation has been reported (Lichtarge et al., 2003; Adikesavan et al., 2011).

### Evolutionary Action Analysis

We run EA analyses (Katsonis and Lichtarge, 2014) for the protein sequences of ERCC2 (XPD), ERCC3 (XPB), ERCC4 (XPF), ERCC5 (XPG), BLM, EXO5, DNA2, RPA1, RPA2, and RPA3 using the corresponding ET score files as inputs. The EA score for each variant is the percentage of functional loss ranging from 0 (benign) to 100 (pathogenic), which were used to identify selection patterns in somatic mutations. For example, an EA score of 80 suggests that the variant has a loss-of-function evolutionary effect of greater than 80% of random amino acid changes in the protein (Katsonis and Lichtarge, 2019). The linear relationships between EA and the fraction of deleterious variants for 5 different proteins shown in Katsonis and Lichtarge (2014) suggest that EA roughly matches the fraction of deleterious variant. For each protein, we compared the scores of the observed mutations in each cancer type with all possible nucleotide substitutions of the query nucleotide sequences [NM_000400 for ERCC2 (XPD), NM_000122 for ERCC3 (XPB), NM_005236 for ERCC4 (XPF), NM_000123 for ERCC5 (XPG), NM_000057 for BLM, NM_022774 for EXO5, NM_001080449 for DNA2, NM_002945 for RPA1, NM_002946 for RPA2, and NM_002947 for RPA3]. The significance *p*-values for this test were calculated with a bootstrap test of 100,000 sampling distributions (Boos, 2003). Notably, the threshold to set EA scores as pathogenic can vary in each gene; in most cases, EA scores >70 are usually observed as pathogenic as reported in TP53 study (Neskey et al., 2015) (also see *Discussion* section). Since most disease mutations in ERCC5 (XPG) and BLM we analyzed have EA scores above 80, we used EA >80 (EA80) as a threshold to focus on the pathogenic (“severe”) mutations in our analyses; EA 30–79 as “mixed” impact mutations; EA <30 as “benign” mutations. All genes (*ERCC2-3-4-5, BLM, EXO5, DNA2*, and *RPA*) with EA scores are listed in Supplementary Table S12. Disease mutations used for the EA analyses are listed in Supplementary Table S13.

### Structural Modeling

We modeled missing loops and domains for full-length BLM and RPA1/2/3 using the RoseTTAFold server (https://robetta.bakerlab.org/) (Baek et al., 2021). The top 5 models were overlaid with crystal structures (PDB: 4O3M for BLM catalytic domain; PDB: 4GOP for RPA1/2/3) to reject severe clash models: e.g., for BLM, residues 1–994 and 640–1417 were modeled independently and then overlaid with PDB 4O3M to select the best model based on RMSD and clashes and combine as a full-length BLM structure with DNA from PDB 4O3M. For RPA, RPA1, RPA2, and RPA3 were modeled independently and then overlaid with the fungal RPA trimer crystal structure (PDB 4GOP) to pick the best model without severe crash (RMSD between 158 pruned atom pairs is 1.110 Å; across all 428 pairs: 4.775 Å). The best model was chosen by examining the clashes and known interactions. DNA was modeled into the best RPA trimer model by superimposing it with the fungal RPA trimer structure (PDB: 4GOP) with some clashes (as expected) on the amino acid side chains on the DNA binding path and Zn-binding motif. We also generated the RPA trimer model using the Rosetta Comparative Modeling (CM) mode (Song et al., 2013) and compared it with the assembled RoseTTAFold RPA trimer model. The assembled RoseTTAFold RPA trimer model was preferred because RPA1 N-ter and RPA2 C-ter aligned well with known PDBs (PDB: 5E7N, RMSD ∼0.7 Å; 4OU0, RMSD ∼0.5 Å), in contrast to the CM model (PDB: 5E7N, RMSD ∼16.8 Å; 4OU0, RMSD ∼8.8 Å). Full-length EXO5 was modeled using comparative modeling with DNA-free EXO5 structure (PDB: 7LW7) in Rosetta Server to preserve the existing crossover-helix structure that was modeled as one long helix by RoseTTAFold modeling. AlphaFold also interestingly predicted a long-helix structure for the cross-over helix and N-terminal region interacting with the nuclease domain, implying a possible regulatory role from the N-terminal region. Without additional modeling of the missing sections of the protein, cryo-EM and x-ray structures were used for XPB and XPD as TFIIH complexes (PDB: 6RO4, 6O9L, and 6O9M), XPF (PDB: 6SXA and 6SXB), and XPG (PDB: 6VBH). DNA was added to the XPG model by overlaying the FEN1 substrate complex (PDB: 5UM9) onto the XPG catalytic domain. Human DNA2 structure from the AlphaFold model (AF-P51530-F1-model_v1.pdb) was used for analyses (Jumper et al., 2021), and DNA was modeled in by superimposition with the mouse DNA2 structure (PDB: 5EAN). The predicted full-length models were deposited in ModelArchive (https://www.modelarchive.org/): XPF-ERCC1 model (doi:10.5452/ma-64cv7); BLM-DNA model (doi:10.5452/ma-n0uwo); RPA-DNA model (doi:10.5452/ma-b0ise).

### Mutation Load Groups

We filtered the CosmicMutantExport.tsv file to select for The Cancer Genome Atlas (TCGA) patient samples, containing a total of 2,427,844 entries. Gene length for the genomic release GRCh38.p13 was obtained from Ensembl (https://useast.ensembl.org/index.html) through BioMart (Ensembl Genes 104) by selecting the “CDS Length” option under “Structures.” From this, we used custom scripts to obtain a list of nonredundant genes, and where a “gene” (ENSG) was defined as the longest “transcript” (ENST) with common ENSGs. The final list contained 19,535 entries. COSMIC gene mutations were intersected with Ensembl gene lengths and normalized per 100 bp per 1,000 patients using custom C++ scripts. Tumor samples were then divided in 3 mutation load groups (MLGs): low, with <41 mutations per sample (2,369 samples, 14,969 genes); medium, with >40 and <701 mutations per sample (6,841 samples, 17,571 genes); high, with >700 mutations per sample (681 samples, 17,533 genes). For each MLG, genes were ranked by normalized mutations (mutations per 100 bp per 1,000 patients) and equally divided into 35 bins. Genes in each bin were then used to conduct a gene set enrichment analysis (GSEA) for each MLG using the Database for Annotation, Visualization and Integrated Discovery (DAVID) v6.8 at https://david.ncifcrf.gov. Gene age was obtained from a published resource (Litman and Stein, 2019).

### Selection Index

A measure of selection for damaging mutations in *ERCC2* in different tumor types was computed as follows: si = (mut*100/tot)/(med/1000), where *mut* was the number of samples with *ERCC2* mutations with EA score ≥60; *tot* was the total number of samples of a tumor type; *med* was the median number of mutations for all samples of a tumor type.

## Results

### ERCC5 (XPG) Alterations Lead to Poor Prognosis in Cancer

As XPG is linked directly to cancer by its roles in NER and R-loop resolution, it has been considered an inhibitor target to block NER for cancer therapy (Kelley et al., 2014). Yet, XPG disease mutations implicate low XPG levels and activity in human disease (Tsutakawa et al., 2020a). We therefore tested whether *ERCC5* (XPG) alterations in terms of simple coding mutations (single base substitutions and small indels) or changes in gene expression in tumors represent a risk factor in cancer.

**FIGURE 1:**
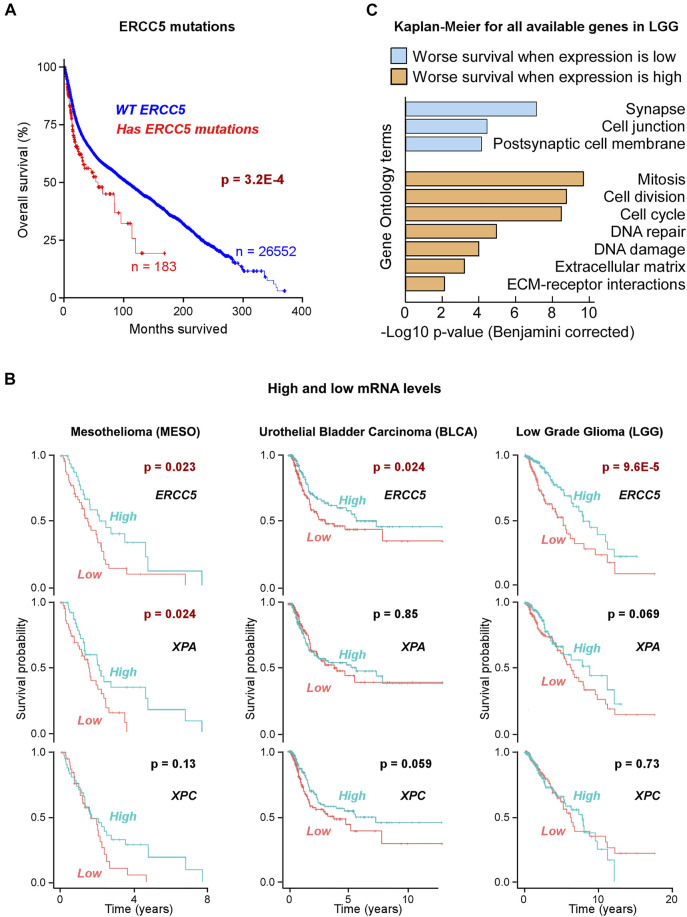
Correlation of *ERCC5* (XPG) expression in tumors with patient survival **(A)** Kaplan–Meier (KM) plot of patients with or without *ERCC5* mutations in their tumors (all cancers), as a function of patient survival after diagnosis. Patients with *ERCC5* mutations in their tumors are at higher risk. Data are in Supplementary Table S1. **(B)** Prognostic risk for tumors with low *ERCC5, XPA,* or *XPC* expression. KM plots of patients with tumors that had *ERCC5, XPA,* or *XPC* mRNA expression levels above and below the mean. Plots are shown for the three cancers with significant prognostic risk based on *ERCC5* expression: mesothelioma (MESO), low grade glioma (LGG), and bladder urothelial carcinoma (BLCA). Only low *XPA* expression in mesothelioma showed significant risk. Data are in Supplementary Tables S2, S3. **(C)** Benjamini–Hochberg-corrected *p*-values and most significant Gene Ontology (GO) terms for gene enrichment analysis of genes identified to yield KM logrank *p*-values <1 × 10^−4^ when expressed at low (below or equal to the mean, blue) or high (above the mean, orange) levels in LGG patients. Data are in Supplementary Table S4.

We first tested if mutations in *ERCC5* were associated with cancer patient survival by analyzing 26,735/42,027 informative patients representing malignancies in >30 tissues from 158 non-redundant studies; of these, 187 patients with *ERCC5* mutations displayed a shorter life span (56.5 median months survival) than 26,552 patients without *ERCC5* mutations (106 median months survival, logrank test *p*-value = 3.2 × 10^–4^) (Figure 1A; Supplementary Table S1). The decreased survival was not due to co-occurring mutations in *TP53*, which were more common (59.3%) than *TP53* mutations alone (32.0%, *p*-value from Fisher’s exact test <0.00001), since patients with *ERCC5* mutations but no *TP53* mutations also displayed poorer outcome (logrank test *p*-value = 5.0 × 10^–5^) than patients without mutations in *ERCC5* and *TP53*. Therefore, we conclude that mutations in *ERCC5* correlate with poor overall survival.

For levels of gene expression, Kaplan–Meier (KM) estimator and hazard ratios (HR) indicated that in 3 out of 33 TCGA tumor types, low (below mean) *ERCC5* expression was a risk factor correlating with poor patient survival. These included low-grade glioma (LGG, 513 patients, HR 95% CI 0.28–0.66, *p*-value 9.6 × 10^–5^; logrank test *p*-value 6 × 10^–5^), mesothelioma (MESO, 85 patients, HR 95% CI 0.30–0.92, *p*-value 0.025; logrank test *p*-value 0.02), and bladder urothelial carcinoma (BLCA, 407 patients, HR 95% CI 0.44–0.95, *p*-value 0.026; logrank test *p*-value 0.02) (Figure 1B; Supplementary Table S2). In LGG, low *ERCC5* expression was also a risk factor (HR 95% CI 0.27–0.86, *p*-value 0.0131) in the absence of *TP53* mutations (51% patients).

As the above ERCC5 (XPG) correlations could point to multiple associations, we tested other NER proteins, e.g., XPA and XPC, and found that they generally did not exhibit the same characteristics in these three cancer types, except for XPA in MESO (Figure 1B; Supplementary Table S3). This observation suggests that although XPG’s role in NER may contribute to the risk factor associated with low expression in tumors, deficiencies in its non-NER functions may also be responsible.

### Downregulation of ERCC5 May be Associated With Poor Cancer Prognosis

Previously, we and others observed that overexpression of DNA repair proteins was associated with poor prognosis (Soll et al., 2017; Thapar et al., 2019). Therefore, we extended the KM estimator to all available genes (∼20,000) in LGG to gain insight into all transcriptomic alterations. At a *p*-value threshold of 1 × 10^−4^, poor survival was seen for 561 out of ∼20,000 genes when expressed at low levels, and for 626 genes when expressed at high levels. The former set was strongly enriched in genes involved in synapse and cell junctions (−log10 *p*-values 7.1 and 4.4, respectively), suggesting an acceleration of tumor cell migration. The latter was related to genes associated with cell division and DNA damage response and repair (−log10 *p*-values 9.7–4.0; Figure 1C; Supplementary Table S4). From the analysis of LGG expression data, DNA repair proteins showed similar *p*-value risk factors if they were overexpressed (Figure 1C; Supplementary Table S4), making XPG low expression unusual as a DNA repair protein.

**FIGURE 2:**
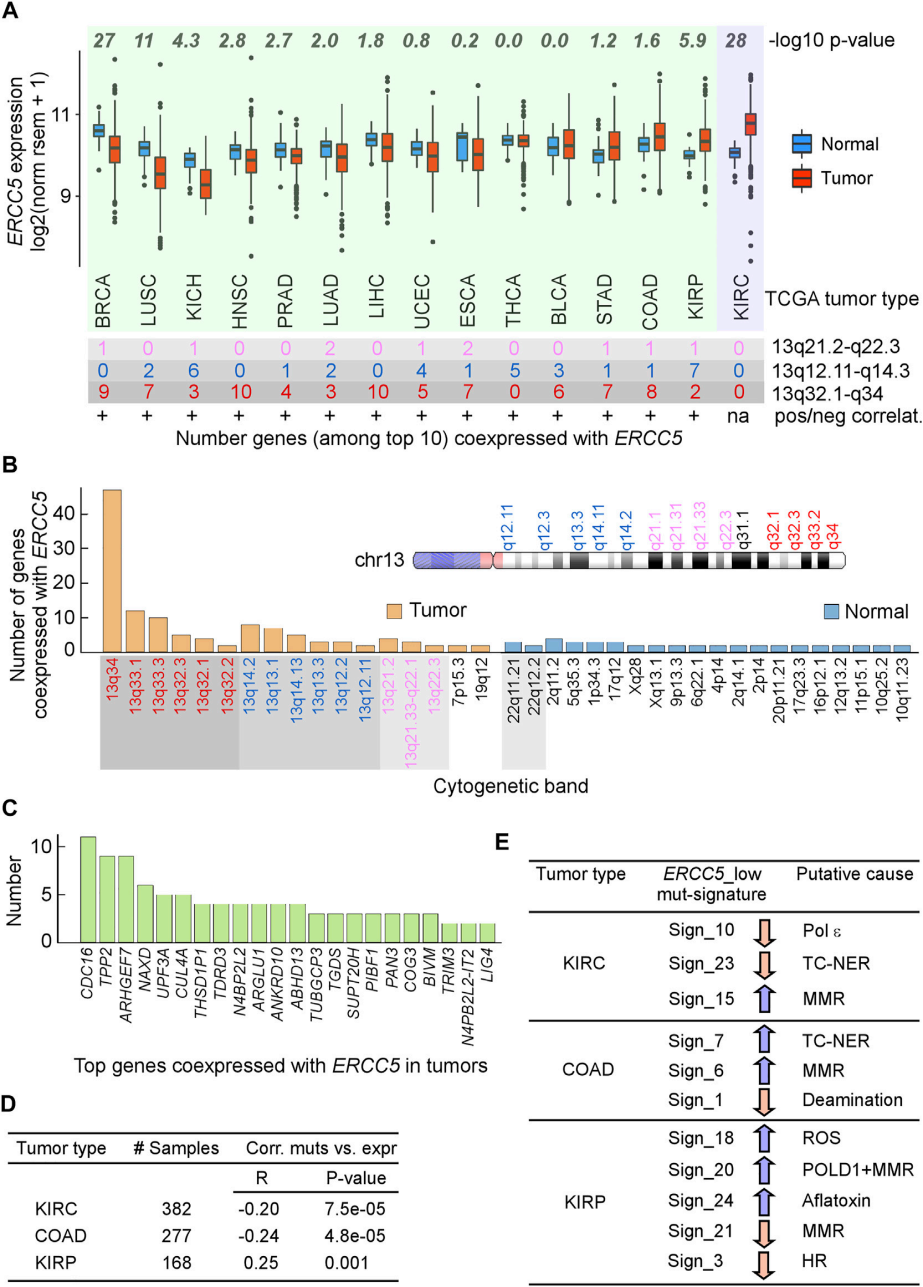
Analysis of ERCC5 (XPG) expression in tumors. **(A)** *Top*, *ERCC5* gene expression in tumors and matched normal tissues from TCGA. *p*-values from Wilcoxon tests. *Bottom*, number of genes out of top 10 whose expression was most highly correlated with that of *ERCC5* in tumors and their location in cytogenetic bands. +, positive correlations; green background, tumors with *ERCC5*-coexpressed genes on chromosome 13; violet background, tumor without *ERCC5*-coexpressed genes on chromosome 13. Data are in Supplementary Tables S5, S6. **(B)** Total number of genes from panel A in each cytogenetic band for tumors and matched controls and cartoon of selected chromosome 13 cytogenetic bands. Data are in Supplementary Table S7. **(C)** Types of genes co-expressed with *ERCC5* in tumors from panel A. Data are in Supplementary Table S8. **(D)** Tumor types that exhibited significant correlations between number of exome-wide simple mutations (single base substitutions and small indels) and *ERCC5* expression. *R*, Pearson’s correlation coefficient; *p*-values from Welch’s *t*-tests. Data are in Supplementary Table S9. **(E)** Samples from Panel D were divided into two groups, one with *ERCC5* expression below (ERCC5_low) and the other with *ERCC5* expression above (ERCC5_high) the median; then, 30 COSMIC mutational signatures (https://cancer.sanger.ac.uk/cosmic/signatures) were compared between the two groups. Arrows indicate whether the ERCC5_low group incurred significantly less (pink) or more (blue) mutations (from Welch’s *t*-tests) than the ERCC5_high group. Signature 10 has been attributed to mutations in polymerase epsilon and defective leading-strand DNA synthesis. Signatures 7 and 23 have been associated with transcription-coupled NER (TC-NER); signatures 6, 15, and 21 have been linked to mismatch repair (MMR) defects; signature 1 has been linked to spontaneous 5mC deamination at CpG sites; signature 18 has been linked to reactive oxygen species (ROS); signature 3 has been linked to defective homologous recombination (HR); and signature 24 has been linked to exposure to aflatoxin. Data are in Supplementary Table S10.

*ERCC5* expression was not elevated in most tumors relative to the corresponding normal tissues. Since low (below the mean) *ERCC5* expression within tumors was associated with increased risk factor, we hypothesized that *ERCC5* expression in tumors might fail to be upregulated in response to increased cell division and DNA damage. Indeed, in 11/15 tumor–normal pairs, *ERCC5* expression was not elevated relative to matched controls (Figure 2A). For most tumors, genes strongly coexpressed with *ERCC5* were located on chromosome 13, where *ERCC5* resides at q33.1 (Figures 2A–C; Supplementary Tables S5–S8). These strong correlations did not appear to arise from copy number alterations, which only occurred in ∼1% of patients (cBioPortal). Of the coexpressed genes (Figure 2C), *NAXD* and *BIVM* also displayed hazard ratios comparable to that of *ERCC5* in LGG, although their association with cancer has not been reported. Kidney renal clear cell carcinoma (KIRC) was noticeably different from the other tumors in that no genes on chromosome 13 correlated strongly with *ERCC5*. Interestingly, KIRC is an outlier as a tumor type where *ERCC5* was highly overexpressed compared to normal cells (Figure 2A), possibly reflecting more DNA damages caused by toxin exposure in kidney. Together, these data suggest that tumorigenesis may enable changes in chromatin structure that redirect *ERCC5* transcriptional control to alternative promoter/enhancers, which may be insensitive to DNA damage response (DDR).

Given that cells derived from XPG knockout mice have increased mutagenesis (Shiomi et al., 2001), an inverse correlation of *ERCC5* expression with mutational frequency would be expected. Therefore, we examined whether *ERCC5* expression correlated with the frequency and mutation spectra in tumors. We identified three tumor types (Figure 2D; Supplementary Table S9), which were also those with higher *ERCC5* expression than in controls (Figure 2A). In colon adenocarcinoma (COAD) and KIRC, *ERCC5* expression was inversely correlated with the number of exome-wide mutations, whereas these two variables exhibited a positive and weak relationship in kidney renal papillary carcinoma (KIRP) (Figure 2D). Furthermore, in COAD and KIRC, but not in KIRP, a significant number of mutations were consistent with defective TC-NER (Figure 2E; Supplementary Table S10). By contrast, KIRC patients with low *ERCC5* exhibited a smaller fraction of TC-NER-associated mutations than patients with high *ERCC5* expression. Together with the observation that *ERCC5* expression was increased in some tumors (Figure 2A) and that coexpression with chromosome 13 genes was absent, these data support the view that, in KIRC, *ERCC5* transcription retains its dependence on DDR. In summary*,* mutations or low expression in *ERCC5* is associated with poor prognosis in some types of cancer, consistent with a need to balance XPG levels for its multiple functions in genome stability.

### Mapping ERCC5 (XPG) Mutation Sites for Disease and VUS

**FIGURE 3:**
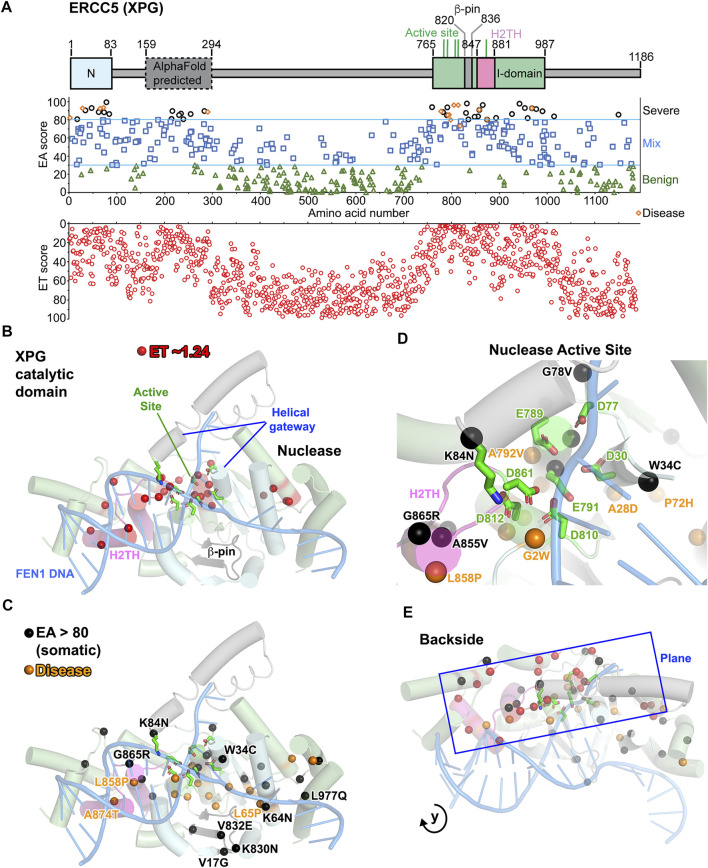
XPG: ERCC5 (XPG) ET, disease, and EA80 VUS. **(A)** *Top*, ERCC5 (XPG) domain schematic; *middle*, disease mutants and VUS mapped with EA scores; *bottom*, amino acid residue mapped with ET scores. **(B)** The most significant residues based on ET scoring, mapped onto a cartoon depiction of XPG catalytic domain (PDB: 6VBH) with substrate DNA model based on FEN1 overlay (PDB: 5UM9). **(C)** Disease and EA80 VUS, mapped onto a cartoon depiction of XPG catalytic domain, as in **(B)**. **(D)** Zoom of nuclease active site, highlighting disease and EA80 mutations. Model and colors are the same as **(C)**. **(E)** Different perspective of XPG catalytic domain cartoon depiction reveals how the plane formed by ET, disease, and EA80 sites extends to the backside of XPG, opposite to the dsDNA binding.

To both test ET scoring and find additional functional sites, we applied ET analysis to XPG. Incising 5′ to the lesion in NER, XPG is a structure-specific endonuclease in the 5′-nuclease superfamily that includes flap endonuclease 1 (FEN1), gap endonuclease 1 (GEN1), and exonuclease 1 (EXO1) (Grasby et al., 2012; Liu et al., 2015; Shi et al., 2017; Tsutakawa et al., 2017). The nuclease catalytic domain, comprising N and I regions separated by over 600 amino acids (aa), has been solved (Figure 3A) (Gonzalez-Corrochano et al., 2020; Mietus et al., 2014; Tsutakawa et al., 2020a) and is similar in structure and function to other 5′ nucleases (Grasby et al., 2012). Based on conservation with FEN1, the active site includes seven mostly invariant carboxylate residues and selects against non-canonical substrates with a two-helix gateway that transitions from disorder to order upon DNA binding (Finger et al., 2012; Patel et al., 2012; Rashid et al., 2017; Sakurai et al., 2005; Tsutakawa et al., 2011). Selection for dsDNA binding includes a H2TH-K^+^ binding site and a beta pin (Figure 3B; Supplementary Video S1). Other regions, predicted to be disordered, may act in DNA contacts and protein–protein interactions critical for NER, BER, and replication-associated repair (Sarker et al., 2005). We identified 23 residues that had an ET score of 2.55, all mapping to the nuclease domain I (Figure 3A; Supplementary Table S11). Relevant to protein conformational dynamics, 7 of the 23 ET most significant residues are proline or glycine. The most significant residues were located along one plane that begins with the H2TH, passes through the nuclease active site and ends up on the backside of XPG-DNA binding site (Figures 3B,E). From modeling how XPG and XPD would bind the NER DNA-bubble, this backside region is likely coordinating excision with XPD (Tsutakawa et al., 2020b).

Mutations in XPG lead to two autosomal recessive genetic disorders, xeroderma pigmentosum (XP) and Cockayne syndrome (CS) (Berneburg and Lehmann, 2001; Bootsma et al., 1995; Fassihi et al., 2016; Lehmann et al., 2018; Okinaka et al., 1997; Scharer, 2008). Ranging from EA scores of 73–96 (Supplementary Table S12), 14 of the 15 disease mutations (9 XP-G and 6 XP-G/CS) mapped to the catalytic domain, which have been shown to destabilize the protein (Tsutakawa et al., 2020a), and all 15 displayed EA scores predicting severe consequences on structure (Figures 3A,C) (Chikhaoui et al., 2019; Emmert et al., 2002; Fassihi et al., 2016; Lalle et al., 2002; Moriwaki et al., 2012; Norris et al., 1987; Nouspikel and Clarkson, 1994; Pugh et al., 2019; Schafer et al., 2013; Soltys et al., 2013; Sun et al., 2015; Zafeiriou et al., 2001). Twelve disease mutations were located on the plane identified in the ET analysis and were located within or at the ends of helices (9 residues) or β strands (3 residues) except two residues 818 and 968 (Figure 3E). Similar to many XP and XP/CS mutations in XPD (Fan et al., 2008; Yan et al., 2019), five mutations (residues 2, 778, 792, 798, and 805) were at the N- or C-terminal ends of helices or β strands. Such ends of secondary structures control tertiary organization, predicted to disrupt local folding upon mutations.

EA80 analysis reveals that 37 VUS were located in the XPG catalytic domain, and over half were located along the same plane revealed by the ET analysis (Figures 3A,E). Four were located in or near the beta hairpin that shifts in response to DNA binding in FEN1 (Orans et al., 2011; Tsutakawa et al., 2011), and two were located where the non-incised DNA strand would bind. Interestingly, seven EA80 mutations outside the nuclease domain were clustered in the region 212–290 aa, predicted to be ordered in the AlphaFold model, including a putative ubiquitin binding motif (Hofmann, 2009; Jumper et al., 2021). This suggests that EA analysis can uncover ordered domains that are not yet determined. Notably, four EA mutations (L65F EA score 70, A874T/S EA scores 80/66, and W968C EA score 93) mapped to the same residues as three XP-G disease mutations (L65P, A874T, and W968C) and would be expected to have a similar pathogenic effect. Two mutations (L858P/I EA scores 91/64) mapped to the same residue as an XP-G/CS disease mutation (L858P); however, the tumor L858I substitution, which adds a β branched side chain to a helix, is a more conservative mutation than L858P CS mutation. Strikingly, most EA80 VUS mutations mapped near the surface, where they could impact DNA and other interactions in the catalytic core, and in functionally important regions. Mutant K84N targeted a catalytically essential helical gateway residue that coordinates the scissile phosphate and shifts it into catalytic position, while E791G altered one of the essential carboxylates in the active site (Figure 3D) (Tsutakawa et al., 2011; Tsutakawa et al., 2017; Tsutakawa et al., 2020a). Potentially identifying a DNA or XPD binding site, EA80 analysis included an invariantly conserved Arg959 (R959S mutation) on the back of XPG, opposite to where the main dsDNA binding would occur, which thus may damage a regulatory site in higher eukaryotes. Among seventeen EA80 mutants (almost half of 37 VUS), three mutants were at the end of secondary structures, eight within secondary structures, and six in a loop region. Thirteen EA80 mutations involved a hydrophobic residue changing to polar or *vice versa* and twelve involved glycines or prolines that are likely to alter XPG conformational flexibility.

These XPG analyses support EA and ET analyses with validation by combined disease mutations and structure. The XPG disease mutations with clear connection to phenotype and protein stability were predicted to be severe according to EA scores. Our TCGA analyses showed that XPG mutations or low expression correlate with worse patient survival, suggesting that mutations that are strongly inactivating/deleterious may be selected against. Thus, it is notable that XPG EA80 mutations represent about 10% of the VUS, underrepresented compared to a theoretical 20% in EA scoring (Katsonis and Lichtarge, 2014). Most of the ET, EA, and disease mutations map to the backside plane of the XPG catalytic domain, suggesting a functional connectivity along this plane plus a previously unrecognized functional site, possibly impacting protein–protein or protein–DNA interactions (Figure 3E).

### ERCC4 (XPF) Predicts Critical Roles in Domain-Interface of Helicase-Like Region

**FIGURE 4:**
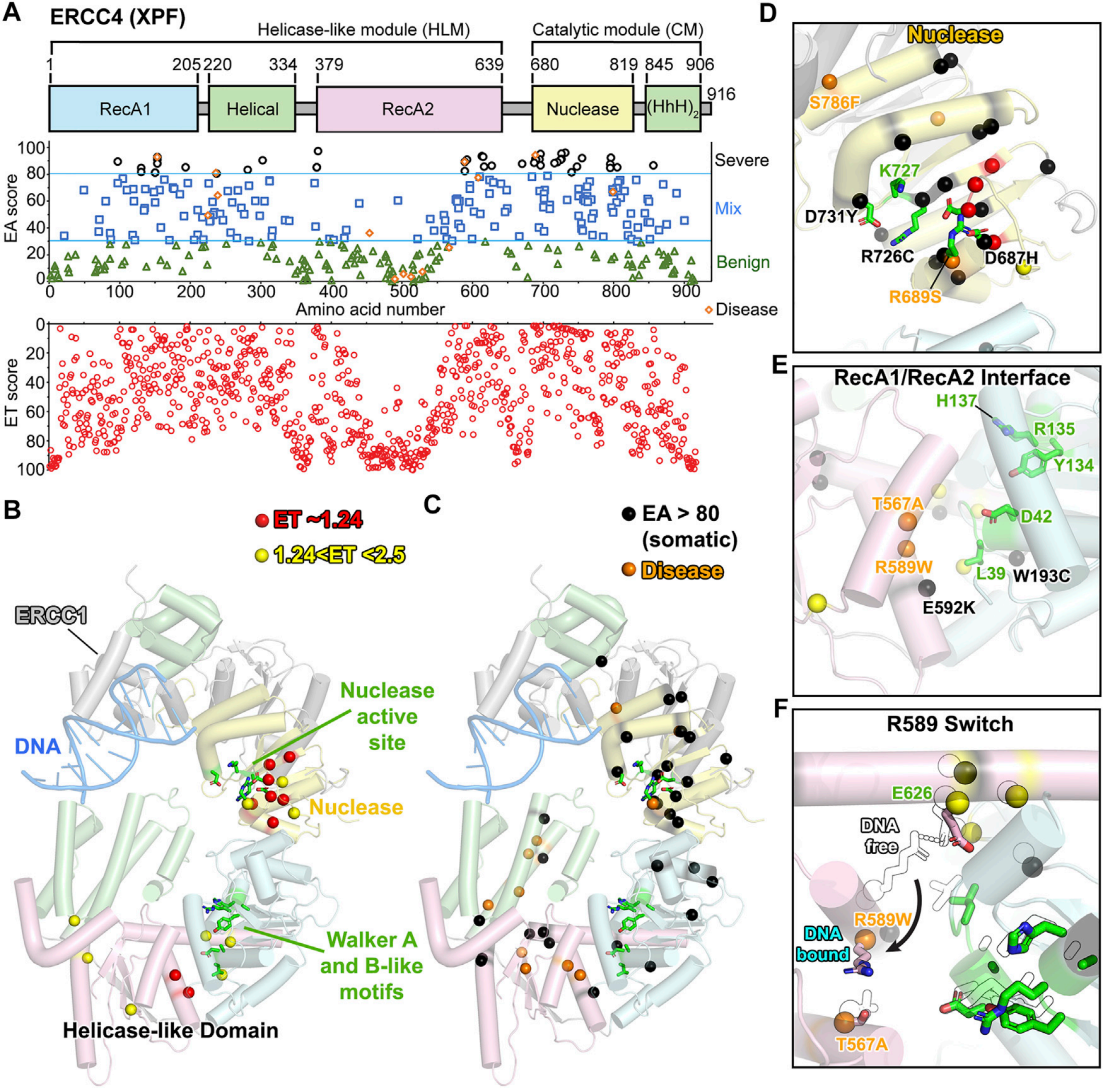
XPF: ERCC4 (XPF) ET, disease, and EA80 VUS. **(A)** *Top*, ERCC4 (XPF) domain schematic; *middle*, disease mutants and VUS mapped with EA scores; *bottom*, amino acid residue mapped with ET scores. **(B)** Two tiers of the most significant residues based on ET scoring, mapped onto a cartoon depiction of XPF-ERCC1 (PDB: 6SXB) with DNA bound by ERCC1 (gray). Domain colors correspond to **(A)**. **(C)** Disease and EA80 VUS, mapped onto a cartoon depiction of XPF-ERCC1, as in **(B)**. **(D)** Close-up view of nuclease active site, highlighting ET-identified, disease, and EA80 mutations. Model and colors are the same as **(B)** and **(C)**. **(E)** Close-up view of Walker A and B-like motifs, highlighting ET-identified, disease, and EA80 mutations. Model and colors are the same as **(B)** and **(C)**. **(F)** Overlay of DNA-free state (PDB: 6SXA, black and white) and DNA-bound state (PDB: 6SXB) of XPF-ERCC1 cryo-EM structures.

In NER, XPF-ERCC1 nuclease incises 5′ to the DNA lesion. Their cryo-EM structures reveal a closed C-shape in the absence of DNA and open when DNA is partially bound to ERCC1 (Jones et al., 2020). XPF-DNA bound structure has not yet been reported. XPF is a two-domain protein, with a nuclease linked to a helicase-like region (Figure 4). The helicase is predicted to be inactive based on the absence of key ATP binding residues in a structural alignment of human XPF with *Pyrococcus furiosus* XPF homolog (PDB: 1WP9) (Nishino et al., 2005). In human XPF, the Walker A motif has a GLGAD instead of GXGK(T/S) in other helicases, and the Walker B motif has YRAH instead of DEA(D/H) (Jones et al., 2020). The significance of the helicase-like region is not yet known, nor the DNA binding by ERCC1.

In our ET analysis, 8 residues have the ET score of 1.23 (1st tier), and 8 had scores between 1.23 and 2.5 (2nd tier) (Figure 4B; Supplementary Video S2). As expected, many of these residues clustered on one side of the nuclease active site. However, half localized in the helicase-like region or more specifically in the RecA2 domain near the interface with other helicase-like domains, suggesting possible domain–domain flexibility with critical roles indicated by low ET residues.

Given its roles in NER, TC-NER, and interstrand crosslink repair, *ERCC4* (XPF) is associated with multiple autosomal recessive diseases, including XP, CS, progeroid syndrome, cerebellar ataxia, and Fanconi anemia (FA). Unlike XPG, the disease mutations of XPF ranged from severe (EA score 94) to mild (EA score 2). Five XP mutations were scored likely benign (Matsumura et al., 1998; Sijbers et al., 1998), and most do not map within the XPF cryo-EM model, because that region is disordered. Notably, we cannot exclude the possibility that these site mutations caused splicing defects, as has been observed in ERCC2 (XPD) (Horibata et al., 2015), suggesting EA analysis may detect such occurrences. Five were mixed (30–70), and five were predicted to be severe (Figure 4A). Patient cells with mutations predicted to be benign had reduced NER activity (Matsumura et al., 1998). Although the XP mutations were spread in their scoring, more severe progeroid syndromes, including XP-F/CS and cerebellar ataxia, exhibited scores from 60 to 93 (Kashiyama et al., 2013; Niedernhofer et al., 2006). Three FA mutations had EA scores from 61 to 94 (Bogliolo et al., 2013; Osorio et al., 2013). These 12 autosomal recessive genetic disease mutations (4 CS or progeroid, 3 FA, and 5 XP) were spread between the nuclease and helicase-like region **(** Figures 4C–E). For FA mutation S786F (EA score 61), the residue is located at the interface between the nuclease and the HhH domain, as first discovered in exonuclease III (Thayer et al., 1995), and the bulkier mutation is likely to disrupt the opening of the C-shape needed for DNA binding (Figure 4D). XP mutation T567A (EA score 25) lines along a hole passage between RecA1 and RecA2 domains. It is difficult to explain why a mutation to alanine would cause a defect, but it is notable that it is within hydrogen bonding range of CS mutation site R589W (EA score 89) (Figures 4E,F). R589 is on a helix that rotates ∼120° from the hydrogen bonding range of T567 in the complex with ERCC1-bound DNA to a salt bridge with E626 in DNA-free state. E626 was scored as significant in the ET analysis (top 1.63%). R589W mutation not only would disrupt packing and salt bridging, but also is likely to impair rotation. Thus, T567A (EA score 25) may impact that allosteric movement that is not picked up in the EA scoring. Notably, R153P (EA score 93), a progeria-causing mutation located in the helicase-like domain at the interface with the nuclease domain, prevents XPF transport into the nucleus (Ahmad et al., 2010), perhaps from proline mutation that disrupted folding of the associated helix.

There were 41 EA80 somatic VUS that scored >80 from 335 identified (12%) and were associated with 34 unique residues (Figures 4A,C–E). Like the disease mutations, the EA80 VUS mapped to both the nuclease and helicase-like regions. However, while EA80 mutations spread throughout the nuclease domain, most of the helicase-like region EA80 mutations mapped near the domain–domain interfaces, significantly consistent with the ET scoring. Three of the tumor-associated mutations (D731Y, R726C, and D687H with EA scores 96, 92, and 89, respectively) were at residues implicated in nuclease activity (Figure 4D). In the helicase-like domain, two EA mutations with EA scores of 91 and 85 located to the RecA1 and RecA2, respectively, exemplify change in charge (E592K) and size (W193C) (Figure 4E) (Enzlin and Scharer, 2002).

In sum, we found that XPF disease mutations have benign to severe EA/ET predictions. Low ET (significant), disease and severe-scoring EA sites not only mapped to the nuclease domain but also to the helicase-like domain, suggesting its significance possibly for structure-specific DNA binding despite a lack in ATP hydrolysis. Those mutations mapped to the interface between RecA1 and RecA2 suggests a conformational change, indicated by comparisons between the DNA-free and DNA-bound XPF-ERCC1 structures (Jones et al., 2020). We suggest that XPF-ERCC1, like other structure-specific nucleases, requires licensing by validating the aberrantly structured DNA substrate before incision may occur, and that licensing entails conformational shifts to allow the scissile phosphate to access the nuclease active site (Dehe and Gaillard, 2017).

### RecA Interfaces of ERCC2 (XPD) and ERCC3 (XPB) Helicases Predicted Critical for Tumor Mutations in TFIIH

**FIGURE 5:**
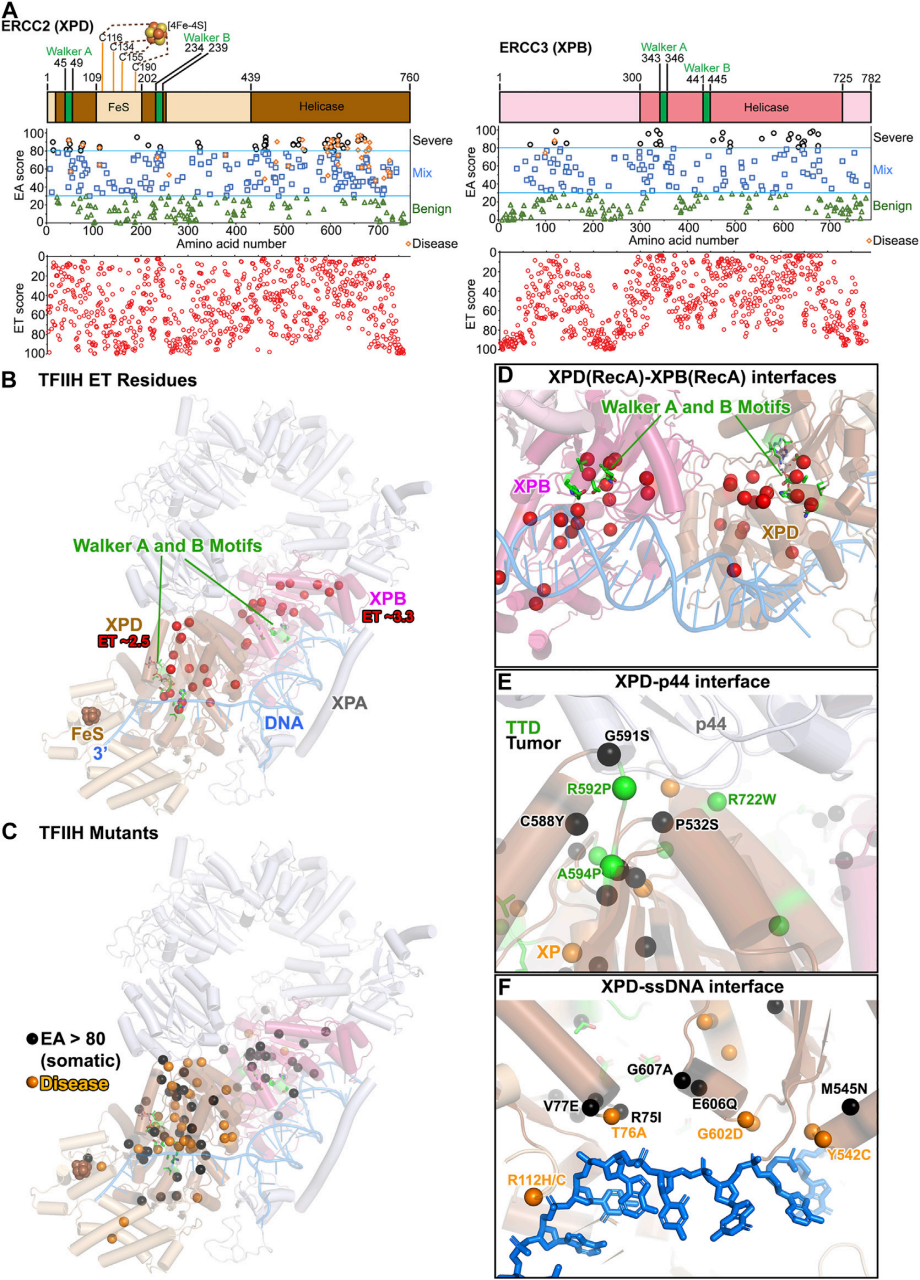
TFIIH: ERCC2 (XPD) and ERCC3 (XPB) ET, disease, and EA80 VUS. **(A)** *Top*, ERCC2 (XPD) and ERCC3 (XPB) domain schematics; *middle*, disease mutants and VUS mapped with EA scores; *bottom*, amino acid residue mapped with ET scores. **(B)** The most significant residues based on ET scoring, mapped onto a cartoon depiction of TFIIH (PDB: 6R04) with XPA and branched DNA. Colors correspond to **(A)**. **(C)** Disease and EA80 VUS, mapped onto a cartoon depiction of TFIIH, as in **(B)**. **(D)** Close-up view of Walker A and B motifs in XPB and XPD, highlighting ET-identified sites. Model and colors are the same as **(B)**. **(E)** Close-up view of the XPD–p44 interface, highlighting disease and EA80 mutations. Model and colors are the same as **(C)**. **(F)** Close-up view of the XPD–ssDNA interface, highlighting disease and EA80 mutations. Model and colors are the same as **(C)**.

In TFIIH (a ten-subunit complex: seven core subunits plus the CAK complex), XPB and XPD act as helicases through dsDNA and ssDNA translocation, respectively (Tsutakawa et al., 2020b). Structures exist for TFIIH in a pre-initiation transcription complex with RNA Polymerase II (RNAPII) and other transcription factors, for TFIIH by itself, and for the TFIIH core subunits with XPA plus pseudo-Y DNA substrate (Greber et al., 2017; Greber et al., 2019; He et al., 2016; Plaschka et al., 2016; Schilbach et al., 2017; Yan et al., 2019). TFIIH core resembles a horseshoe with XPB and XPD located at the ends that lie next to each other (Figure 5B; Supplementary Video S3). Both have RecA1 and RecA2 helicase domains, with the Walker A and B motifs at the interface (Figure 5A). XPD also contains an iron-sulfur (FeS) domain and an arch domain, which are predicted to open for ssDNA binding (Fan et al., 2008; Liu et al., 2008; Wolski et al., 2008). The role of FeS cluster is postulated to communicate with other FeS-containing proteins through charge transfer along DNA (Mui et al., 2011; Sontz et al., 2012; Fuss et al., 2015).

Mapping exclusively to the translocase domains, 19 XPB and 16 XPD residues were deemed most important, with ET scores of 3.3 and 2.5, respectively (Figure 5B). Most located to the DNA binding RecA2-RecA1 interface, consistent with the ATP-induced motion of these DNA interfaces being critical for function (Figure 5D). Except for one XPD residue (R683) at the DNA binding interface, the others mapped at interfaces with other TFIIH subunits: the p44 and p62 interfaces with XPD, and the p8 and p52 interfaces with XPB. These residues identified in the ET analysis support the significance of dynamic communities of residues that move together, as suggested from computational studies (Yan et al., 2019). More residues at the DNA interface, close to the XPB–XPD interface and the FeS site, were in the 2nd tier of ET including 15 XPD residues with ET scores between 2.5 and 5 and 27 XPB residues with scores between 3.3 and 6.6. Interestingly, no residues at the MAT1 interface were identified in the 1st or 2nd tier, consistent with the notion that MAT1 locks down XPD during transcription or in solution but does not have a mechanistic role (Tsutakawa et al., 2020b).

Unlike XPG where disease mutations were mostly predicted to be severe by EA analysis, the EA scores of the 14 XP-D, 8 XP-D/CS, and 16 TTD disease mutations ranged from 53–92, 52–92, and 50–96, respectively (Figures 5A,C). In general, scores of 30–70 are considered mixed, with protein-dependent impacts. The TTD mutations mapped at or close to interfaces, suggesting defects in TFIIH assembly (Figure 5E). TTD mutation R722W in XPD has been reported to abrogate p44 interaction (Coin et al., 1998). Likewise, disease mutations (XP-D/CS/TTD A725P/T/V, TTD R592P) and somatic mutations (G591S, P532S) would disrupt the interaction with p44. As XPD helicase activity is known to be stimulated by p44 (Coin et al., 1998; Kuper et al., 2014), weakening the XPD–p44 interface by mutations would negatively impact TFIIH regulation for NER and transcription (Kuper et al., 2014). Although XPD helicase function is dispensable during transcription, its existence in TFIIH is essential to interact with MAT1 protein in CAK kinase module that regulates transcription. This critical interaction is highlighted by another TTD mutation, C259Y, which is located at the Arch domain of XPD and could cause the partial unfolding of this domain, leading to defects in transcription and cell cycle regulation (Abdulrahman et al., 2013). The XP mutations mapped primarily to the DNA binding interface or the Walker motifs, likely disrupting XPD helicase activity. CS mutations mapped within the dynamic communities determined computationally, suggesting defects in TFIIH movements. For XPB, there were only two disease mutations, one XP-B/CS (F99S, EA score 75) and one TTD (T119P, EA score 88). These two mutations did not localize in the XPB helicase domain (Fan et al., 2006), but in a region connecting XPB to p52 subunit. Like XPG, EA scores were ineffective in differentiating among the different phenotypes of the diseases. Notably, XPD forms a major interface with RNAPII during transcription, and a destabilized XPD structure will disrupt RNAPII-XPB connection (Yan et al., 2019; Tsutakawa et al., 2020b). The milder scoring mutations in XPD and the paucity of XPB disease mutations may reflect the need for TFIIH structural integrity and essential role of XPB in transcription.

In contrast to the 42 disease mutations mapping to XPD RecA domains and only 2 in XPB, the EA80 cancer VUS mapped to both XPB and XPD helicase regions more evenly (Figures 5A,C). There were 49 EA80 mutations in XPD out of 326 (15%, mapping to 40 unique residues) and 29 in XPB out of 251 (12%, mapping to 25 unique residues). Many mapped to the RecA1–RecA2 interface (Figure 5C), to the protein–protein interaction interface (Figure 5E), and to the DNA binding interface (Figure 5F), and therefore are likely to disrupt both transcription and NER. Given that transcription is generally upregulated in tumors, it will be interesting to determine how these mutations may be exploited by tumor cells to support their growth.

In sum, our ET, disease, and EA analyses highlight the significance within the TFIIH complex of the interface between the RecA1 and RecA2 subdomains, the DNA binding interface, and the interface with other subunits, which supports and extends previous observations (Tsutakawa et al., 2020b). What sets TFIIH apart from other proteins in this study was the difference in mutation location in XPB and XPD. Disease mutations were primarily in XPD with only two in XPB, while EA80 tumor mutations localized more evenly in XPB and XPD. As not all proteins are associated strongly with disease mutations, this difference between disease and tumor mutations could only be detected by considering XPB and XPD together within the context of TFIIH. These results highlight that the survival requirements are distinct between disease and tumor mutations and merit consideration in this type of analysis.

### The Most Severe BLM Missense Mutations are Predicted to Reduce the Helicase Domain Stability

**FIGURE 6:**
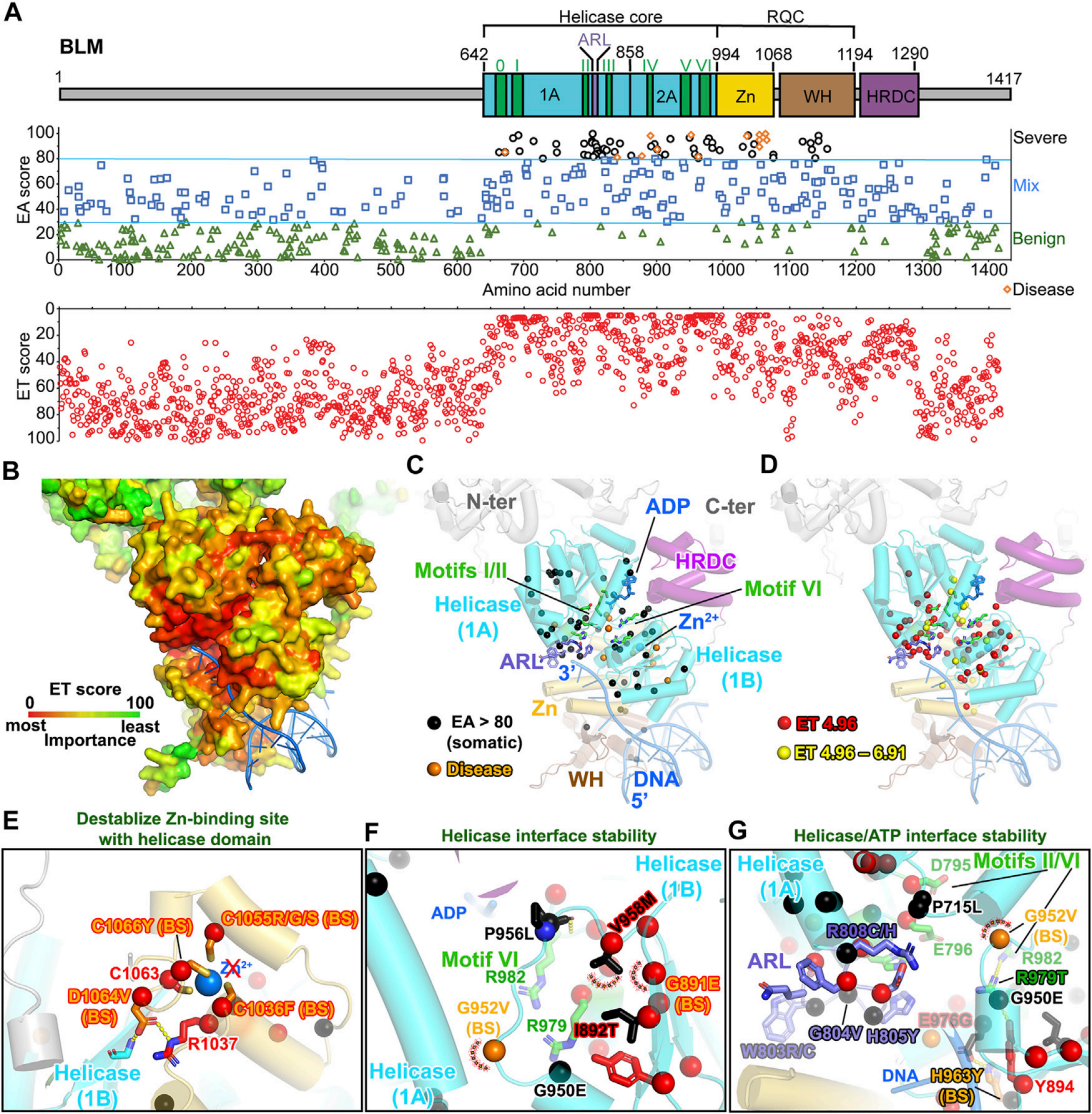
BLM helicase domains are the main target of EA80 VUS. **(A)** *Top*, BLM domain schematic; *middle*, disease mutants and VUS mapped with EA scores; *bottom*, amino acid residue mapped with ET scores. **(B)** ET mapping on BLM structure (PDB: 4O3M + RoseTTAFold) in surface presentation. DNA is shown in blue ribbon. **(C)** Disease mutation and EA80 VUS (orange and black spheres) are mapped on BLM structure shown in cartoon depiction. Domains are colored, corresponding to **(A)**. **(D)** The most significant residues (red and yellow spheres) based on ET scoring are mapped onto the BLM structure. **(E–G)** Close-up view of impacts of disease and EA80 VUS on helicase domain interfaces of BLM. Sphere and sticks are colored according to EA/ET on **(C)** and **(D)**. Overlapping EA/ET/disease residues are shown in their corresponding colors. BS, Bloom syndrome.

BLM helicase, a RecQ-family helicase, unwinds duplex DNA in a 3′–5′ direction *via* ATP hydrolysis and plays key roles in regulating DNA replication, repair, and recombination (Kaur et al., 2021). The core domain contains two RecA-like ATP binding domains, followed by a RQC (RecQ family-specific C-terminal domain) domain, containing a Zn-binding subdomain and a WH (winged helix) DNA binding subdomain that is responsible for strand separation by a β-hairpin (Figure 6A) (Swan et al., 2014; Newman et al., 2015). The HRDC (helicase and RNase D C-terminal) domain is required for specifically binding and unwinding double Holliday junctions (Wu et al., 2005). Finally, the N- and C-terminal flexible regions are proposed to interact with protein partners, including RPA, Topoisomerases, RMI1, and RAD51 (Doherty et al., 2005; Yin et al., 2005; Plank et al., 2006; Bugreev et al., 2007; Bythell-Douglas and Deans, 2021). Our EA analyses revealed that all the Bloom syndrome (BS) disease mutations, including functional polymorphism variants, were scored above 80 (Figure 6A; Supplementary Table S12). Together with severe somatic mutations (EA >80), they are primarily located in helicase core and RQC domain. This observation is reinforced by ET analysis showing that the helicase core domain and Zn-binding subdomain rank in functional importance above the WH and HRDC DNA binding subdomains or the disordered protein–protein interaction regions on N- and C-termini (Figures 6A,B).

Strikingly, BLM contains over 1,000 germline VUS; we therefore included them in our analysis to evaluate if the functional sites are selected using EA >80 and top 2-tier ET. Our EA80 results showed 118 germline VUS out of 1,021 (∼11.5%, 115 unique residues) and 74 somatic EA80 VUS out of 692 (∼10.8%, 50 unique residues) (Figures 6A–D; Supplementary Figure S1; Supplementary Table S12; Supplementary Video S4). Of somatic mutations, P868L occurs 59 times and V1321I occurs 62 times. Interestingly, 49 residues out of 59 ET (1st tier) are exclusively located in the helicase domain; the others are located in the Zn-binding subdomain, most heavily on the Zn-binding site, which also contains 3 disease mutations on conserved cysteines (Guo et al., 2007). Notably, BS variants abolish Zn-binding with cysteine mutants (C1036F, C1055R/G/S, and C1066Y, EA 89–99) that have been shown to decrease DNA binding, resulting in lower helicase activity (Figure 6E) (Guo et al., 2005), consistent to other RECQ-family helicases (Ren et al., 2008). Most EA80 somatic and germline VUS are located on helicase domains, which impact ATPase activity; the rest are on Zn-binding and WH domains, which are critical for DNA binding and strand separation. The EA80 somatic and germline VUS destabilize the ATPase domains that impact ATP binding and helicase activity (Figures 6F,G; Supplementary Figures S1B,C). We conclude that the BLM helicase domains are the primary mutational targets in both cancer and the germline, with predicted impact on its helicase activity. Interestingly, a highly conserved 8-residue aromatic-rich loop (ARL) in the helicase 1A/2A subdomain interface serving as a sensor for DNA binding coupling to ATP hydrolysis function had high EA scores (4 cancer mutations with EA of 83.17–99.68 and 2 germline mutations with EA of 83.17–94.01) and low ET scores (7 with ET of 4.96), suggesting its functional importance (Zittel and Keck, 2005). Two residues W803(to R/C) and R808 (to C/H) are overlapped in both EA of somatic and germline and ET analyses, implying that ARL is a potential hot spot for mutations (Figure 6G). Mutant W803R showed total loss of function, and R808L equivalent mutant in *E. coli* RecQ helicase significantly reduced helicase activity (Zittel and Keck, 2005; Mirzaei and Schmidt, 2012). Together, our EA/ET analyses show that mutations are predicted to impair BLM helicase function, resulting in defects of DNA repair, replication fork recovery, replication stress, and eventually genome instability. Thus, cancer cells overexpress BLM to increase their survival, which in turn has been linked to poor cancer prognosis (Hambarde et al., 2021).

### The Most Severe EXO5 Missense Mutations are Predicted to Reduce Structural Stability

**FIGURE 7:**
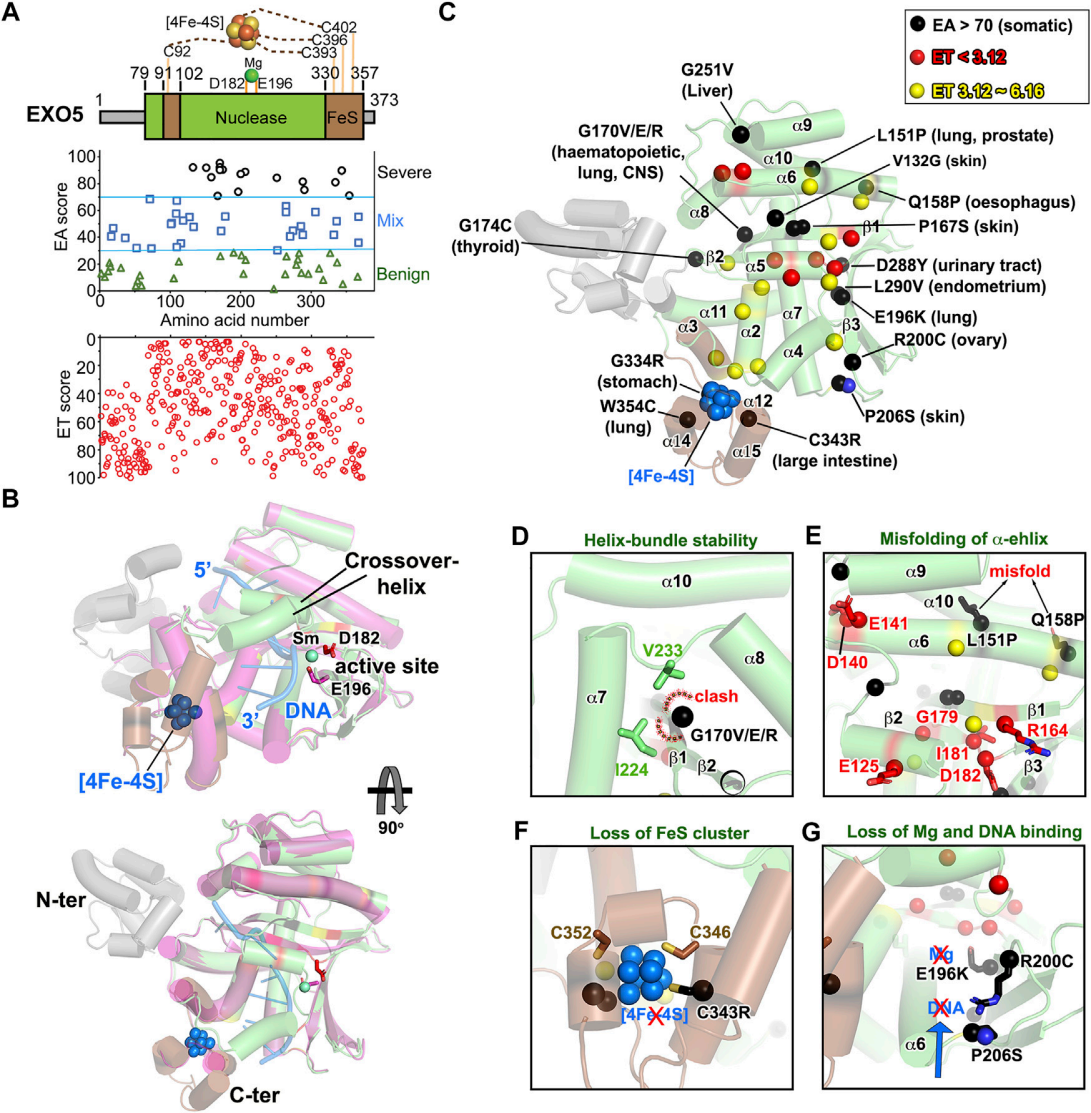
EXO5 nuclease structure folding is impacted by EA70 VUS. **(A)** *Top*, EXO5 domain schematic; *middle*, disease mutants and VUS mapped with EA scores; *bottom*, amino acid residue mapped with ET scores. **(B)** Structure overlay of DNA-free (PDB: 7LW7) and DNA-bound EXO5 (PDB: 7LW9). Metal binding site is shown as sticks on D182 and E196. **(C)** ET and EA70 (shown in red, yellow, and black spheres) mapping on the EXO5 structure with corresponding tumor sites. **(D–G)** Close-up view of impacts of EA70 VUS on structure folding and the DNA/metal binding site of EXO5.

EXO5 is a recently discovered structure-specific nuclease for stalled replication fork restart (Hambarde et al., 2021). EXO5 resects only ssDNA with open-end from 5′ to 3′ direction, which is enforced by RPA (Sparks et al., 2012). EXO5 folds into a single nuclease domain with a [4Fe-4S] cluster bound region connecting N- and C-terminal conserved cysteine residues (Figure 7A; Supplementary Video S5). The ssDNA substrate enters through a channel near the FeS cluster region and exits under a crossover helix (Figure 7B). The active site residues D182 and E196 are located at the center of splayed apart β-sheets. We set EA >70 as a cutoff to examine the impact of cancer mutations based on the E196K active site mutant score (EA ∼74), which remains in the “pathogenic” EA score range 70–100. There are 20 EA70 mutations out of 85 (∼23.5%, 17 unique residues), and no disease mutations have been reported yet (Figures 7A,C). Residue G170, located on the β1-strand between α6-helix and β2-strand, was frequently targeted with highly damaging substitutions (G170V/E/R, EA ∼95/92/90) expected to impact the neighboring α-helix bundle and cause misfolding/structure destabilization (Figure 7D). The misfolding of EXO5 is also induced by L151P (EA ∼92) and Q158P (EA ∼85) on the α6-helix (Figure 7E). Interestingly, L151P mutant was also found in three prostate cancer families where it affected all siblings, impairing homology-directed repair and nuclease activity due to misfolding (Ali et al., 2020). The low ET (most important) residues (E125, R164, I181, and D182) are located at crossover-helix and β2-stand near the active site, suggesting their critical relationship for nuclease activity (Figures 7C,E). E125 is absolutely conserved on the crossover-helix, which may play a role in regulating activity as implied by H121A and R124A mutants on the crossover-helix that abolished nuclease activity, but not DNA binding (Hambarde et al., 2021). The E140 and D141 (ET ∼ 3.12) located at the beginning of α6-helix were predicted to be important for four helix-bundle (α6, α8–10) stability and 5′-end ssDNA binding stability (Figures 7B,C). Furthermore, one of the four conserved cysteine mutants, C343R (EA ∼90), which ligates to the FeS cluster, damages the integrity of the FeS cluster region to impair nuclease activity (Figure 7F) (Sparks et al., 2012). DNA and metal binding mutants, R200K (EA ∼87) and E196K (EA ∼74), respectively, would significantly decrease nuclease activity (Figure 7G), consistent with and extending published work (Sparks et al., 2012; Hambarde et al., 2021). These results suggest that the cancer-associated missense mutations destabilize EXO5 structure, which will impair its nuclease activity likely reducing replication fork restart and resulting in alternative origin firing and instability.

### Severe Cancer Mutants are Clustered in DNA2 Helicase Domain

**FIGURE 8:**
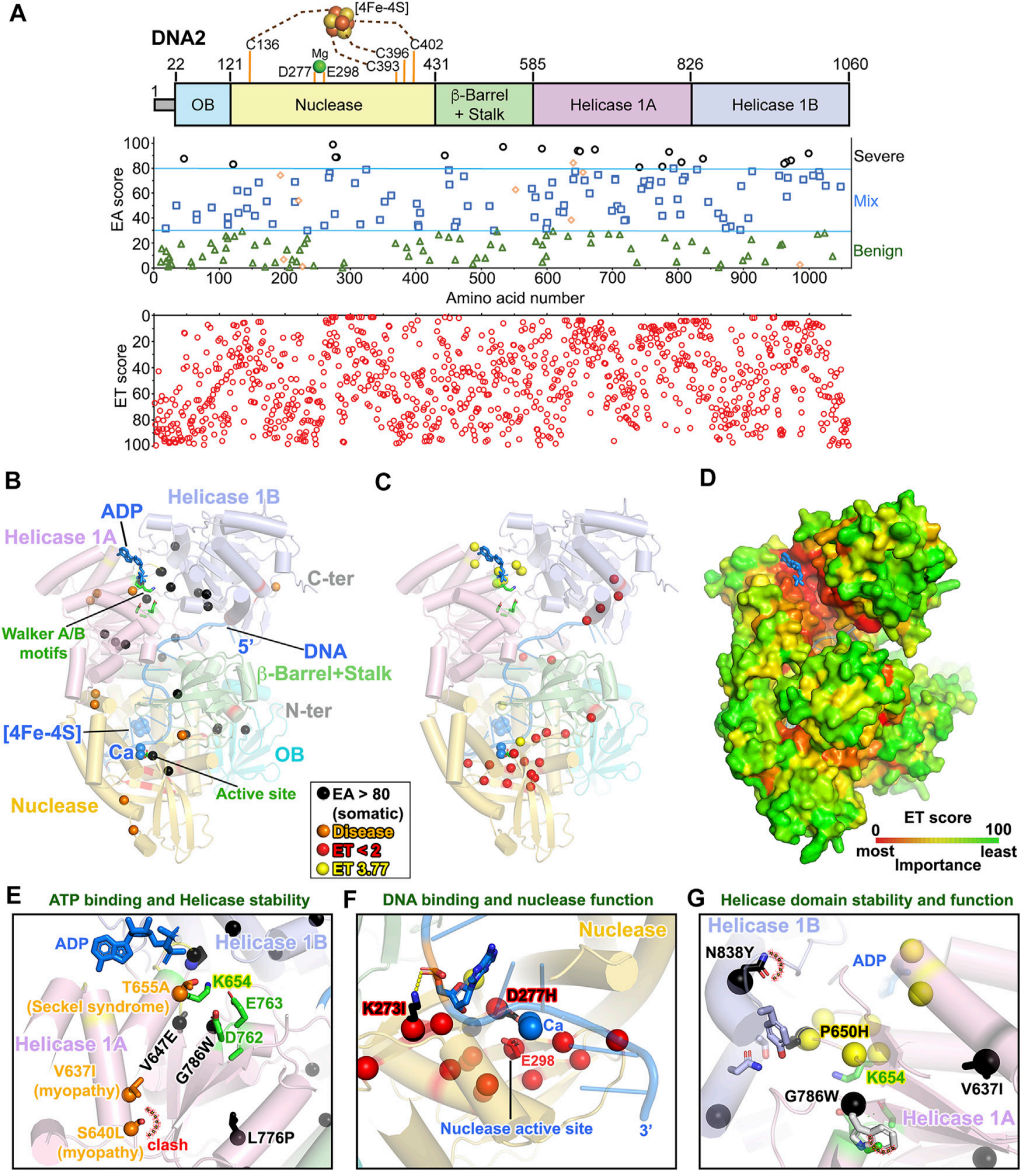
EA80 VUS are mostly clustered at DNA2 helicase domain interface. **(A)** *Top*, DNA2 domain schematic; *middle*, disease mutants and VUS mapped with EA scores; *bottom*, amino acid residue mapped with ET scores. **(B)** Disease mutation and EA80 VUS (orange and black spheres) are mapped on DNA2 structure (AlphaFold + DNA/Ca/FeS/ADP from PDB: 5EAN) shown in cartoon depiction. Domains are colored, corresponding to **(A)**. **(C)** The most significant residues (red and yellow spheres) based on ET scoring are mapped onto the DNA2 structure. **(D)** ET mapping on the DNA2 structure in surface presentation. DNA and ADP are shown as blue ribbon and sticks, respectively. **(E–G)** Close-up view of impacts of disease and EA80 VUS on helicase/nuclease domains of DNA2. Spheres and sticks are colored according to EA/ET on **(B)** and **(C)**. Overlapping EA/ET/disease residues are shown in their corresponding colors.

DNA2 is a structure-specific 5′–3′ nuclease/helicase that contains a PD-(D/E)XK superfamily nuclease motif resembling EXO5 plus a superfamily 1 (SF1) helicase domain (Figure 8A). DNA2 acts in DNA double-strand break repair (Zhu et al., 2008), Okazaki fragment maturation (Bae et al., 2001), and stalled replication fork restart (Hudson and Rass, 2021; Thangavel et al., 2015; Zheng et al., 2020). The nuclease and helicase domains are connected by a β-barrel domain with a stalk of two long α-helices to form a DNA binding tunnel for threading (Figure 8B; Supplementary Video S6). The N-terminal OB-fold domain is packed against the nuclease domain and interacts with RPA1 NAB domains (Zhou et al., 2015).

The nuclease and helicase activities of DNA2 are mutually coupled as nuclease function strongly limits its helicase activity by degrading 5′-ssDNA tail, but at the same time helicase activity promotes efficient degradation of long stretches of ssDNA (Levikova et al., 2013; Levikova et al., 2017). Thus, nuclease-dead mutant E675A in yeast (E298 in human) promotes helicase unwinding activity, and helicase-dead mutant K1080E in yeast (K654 in human) causes DNA-end resection defects (Levikova et al., 2017; Miller et al., 2017), suggesting that DNA2 nuclease activity is critically coupled to helicase function to keep up with its helicase partners such as BLM or WRN on DNA-end resection. Interestingly, the FeS cluster coordinating the N-terminal and C-terminal cysteine residues of the nuclease domain abolishes ATPase activity (Pokharel and Campbell, 2012), supporting the crosstalk between nuclease and helicase domains. Strikingly, in our EA analysis, most EA >80 somatic mutations (∼10%, 23/230, 19 unique residues) are clustered in helicase domain, but disease mutations are scattered in both nuclease and helicase domains (Figures 8A,B).

The 1st tier of ET analysis showed that the most important residues were located on the β-strands of nuclease active site and on the tips of helices (R944, Q946, N913, and E916) of helicase 1B subdomain where 5′-DNA is located, implying a previously unrecognized functional site. The 2nd tier of ET clustered near the ATP binding site (Figures 8C,D). Interestingly, mapping the whole ET scores in the entire DNA2 structure reveals that important functional sites are mostly buried (Figure 8D). Two low ET residues E451 and W444 on the long stalk are at the interface with the helicase 1A subdomain (Figure 8C), and W444 was mutated to leucine (EA ∼90) in EA80 VUS, suggesting the critical function of the stalk to helicase activity. Notably, some mutations with low EA scores are also associated with disease, where some patients developed a mild weakness of limb-girdle muscles, but some with more progressive myopathy (Ronchi et al., 2013). Residues R198H (EA ∼6.68) and K227E (EA ∼1.08) located in the nuclease domain reduced both nuclease and helicase activity significantly, likely impacting the stability of nuclease–helicase interface and helix-capping. In contrast, V637I (EA ∼38.26) located in the helicase domain (Figure 8E) results in higher helicase activity but lower nuclease activity revealing coupling effects between nuclease and helicase functions.

Disease mutants T655A (EA ∼76.56) and S640L (EA ∼86.17) found in patients with Seckel syndrome and severe mitochondrial myopathy are located near the ATP binding site K654 and impact the helicase domain 1A stability (Figure 8E) (Ronchi et al., 2019; Tarnauskaite et al., 2019), causing defects in replication fork recovery. Moreover, somatic mutant K273I (EA ∼99) would be expected to decrease DNA binding significantly, and residue D277H (EA ∼89) would cause the loss of nuclease activity (Figure 8F), as the D277A nuclease-dead mutant (Zhou et al., 2015). Both residues are also associated with low ET (most important residues). Furthermore, the highly clustered EA and ET regions near the ATP binding site reveal the possible loss of helicase activity due to clash and destabilization between the interface of helicase 1A and 1B subdomains by mutants P650H (EA ∼94), G786W (EA ∼93), and N838Y (EA ∼88) (Figure 8G).

Our EA analyses indicate that most severe pathogenic somatic mutations are mostly in the helicase domain. Indeed, DNA2 helicase activity is required for replication fork recovery, suggesting that helicase function is essential for resolving toxic replication intermediates into 5′-flaps for removal by its nuclease function (Olmezer et al., 2016; Appanah et al., 2020). Hyperactive nuclease activity of DNA2 with compromised helicase function could not efficiently degrade long stretches of 5′-ssDNA, as indicated in helicase-dead mutant experiments (Levikova et al., 2017; Miller et al., 2017). Therefore, mutations impairing DNA2 helicase/nuclease would result in genome instability and promote tumorigenesis. Yet, many tumors upregulate DNA2 helicase/nuclease for their survival to resolve replication stress (Peng et al., 2012; Strauss et al., 2014).

### Severe RPA Mutations Imply Structural and Assembly Defects

**FIGURE 9:**
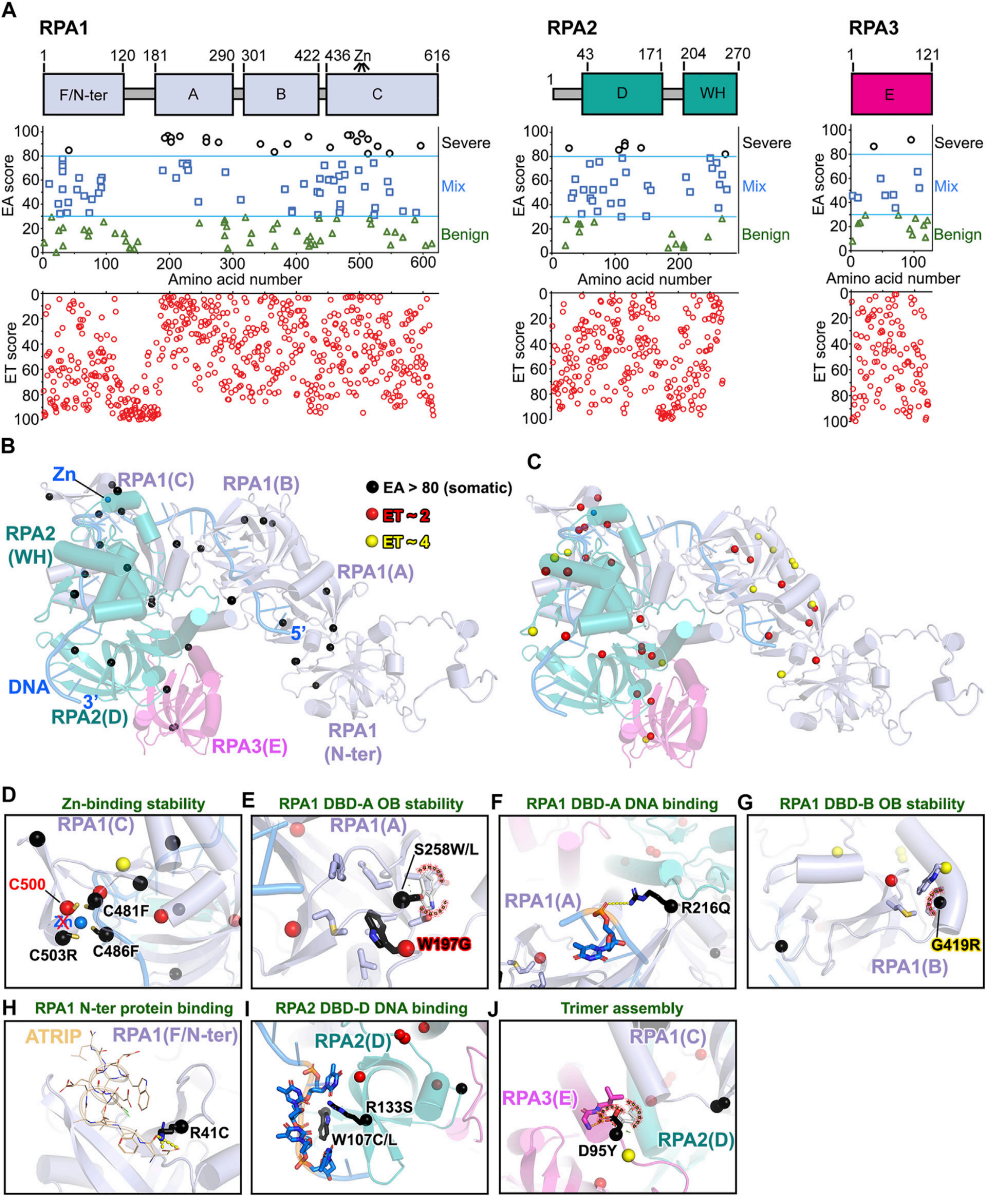
EA80 VUS of RPA impact OB-folds. **(A)** *Top*, RPA1-2-3 domain schematics; *middle*, disease mutants and VUS mapped with EA scores; *bottom*, amino acid residue mapped with ET scores. **(B)** EA80 VUS (black spheres) are mapped on RPA structure (RoseTTAFold + DNA/Zn from PDB: 4GOP) shown in cartoon depiction. Domain colors correspond to **(A)**. **(C)** The most significant residues (red and yellow spheres) based on ET scoring are mapped onto the RPA structure. **(D–J)** Close-up views of impacts of EA80 VUS on OB-folds, DNA binding, and trimer assembly of RPA. Sphere and sticks are colored according to EA/ET on **(B)** and **(C)**. Overlapping EA/ET/disease residues are shown in their corresponding colors.

RPA is a ubiquitous ssDNA binding protein that is essential for genome stability (Bhat and Cortez, 2018). It forms a heterotrimer consisting of RPA1 (∼70 kDa, aka RPA70), RPA2 (∼32 kDa, aka RPA32), and RPA3 (∼14 kDa, aka RPA14) (Figures 9A,B; Supplementary Video S7) (Fanning et al., 2006; Wold, 1997). We used RoseTTAFold modeling to predict full-length human RPA trimer (see *Materials and Methods* section), DNA was superimposed using the fungal RPA trimer structure (PDB: 4GOP). While the DNA location is the same, the RPA trimer structure shifts (RMSD ∼3.47 Å, aligned 396 to 396 atoms) with some DNA clashes. The oligonucleotide/oligosaccharide-binding (OB) folds of the DNA-binding domains (DBDs) A, B, C, and D are responsible for tight ssDNA binding affinity (*K*_d_ ∼50 nM) (Brosey et al., 2013; Kim et al., 1994), which are predicted to be mostly impacted by somatic mutations based on our EA analysis [EA >80, RPA1 ∼22% (26/161, 23 unique residues); RPA2 ∼11% (6/55, 6 unique residues); RPA3 ∼12.5% (3/24, 2 unique residues)] (Figures 9A,B **)**. Surprisingly, the most important (low ET scores) residues also emphasize the OB-fold stability instead of DNA binding path (Figure 9C). For example, DBD-C of RPA1 contains a zinc-finger motif with four-conserved cysteines. Deletion of the Zn-finger motif has negligible impact on ssDNA binding (Kim et al., 1996) but is essential to stabilize the domain *via* redox regulation for ssDNA binding (Bochkareva et al., 2000; You et al., 2000). The Zn-finger motif is required for DNA replication and MMR, although not for NER (Lin et al., 1998). Strikingly, the top EA scores ranked three of the four conserved cysteines (C503R, EA ∼98; C481F, EA ∼97; C486F, EA ∼97) as the most severe mutations (Figure 9D), suggesting that Zn-finger motif mutations impede ssDNA binding during the replication stress response, which may cause tumorigenesis.

As the DBDs of RPA are critical for ssDNA binding to form a functional complex, we found that most EA80 mutations are located in DBD-A, B, C, and D. Mutants W197G (EA ∼97) and S258L/W (EA ∼95/92) could destabilize the OB-fold of DBD-A (Figure 9E), so could the G419R (EA ∼96) for DBD-B (Figure 9G). Interestingly, these mutants and low ET (most important) residues (Figure 9C) are far away from the DNA binding path, suggesting potential functional roles that allosterically modulate ssDNA binding ability. While R216Q (EA ∼96) on DBD-A and W107C/L (EA ∼92/89) and R133S (EA ∼87) on DBD-D may allosterically impair DNA binding (Figures 9F,I), R41C (EA ∼85) on RPA1 N-terminal domain could not as this domain functions by interacting with protein partners (Xu et al., 2008), so mutations would most likely weaken partner protein binding (e.g., with ATRIP) (Frank et al., 2014) (Figure 9H). Moreover, mutant D95Y (EA ∼92) on RPA3 located on RPA trimerization helix core was also picked up by our EA analysis. This mutant would disrupt the trimerization, which regulates switching from 8 to a 30-nucleotide binding mode, resulting in reduction of regulation by RPA OB folds and DNA binding interface (Bochkareva et al., 2002) (Figure 9J). Destabilization of OB-folds could compromise the DBD’s ability to bind ssDNA and form a functional complex. For example, L221P mutation in RPA1 DBD-A dramatically reduced ssDNA binding and lost ability to localize at DNA damage sites, resulting in DNA repair defect despite its ability to form a trimer with RPA2 and RPA3 (Hass et al., 2010). The phenotype of this mutant causes high tumor progression when heterozygous and is lethal in mice when homozygous, indicating the importance of RPA DBD structure stability.

As RPA binds to DNA in a dynamic manner, each DBD is critical to coordinate the DNA binding between functional states with its protein partners (Yates et al., 2018). It is challenging to analyze dynamic proteins like RPA, but it can be complementarily characterized by solution techniques such as small-angle x-ray scattering (SAXS) combined with molecular-dynamic simulation computational method to define conformation and flexibility of mutant proteins (Pretto et al., 2010; Brosey et al., 2013; Brosey and Tainer, 2019). DNA binding affinity and cell-based functional assays would be useful to test the selected high EA (70–100) scored mutants.

### Mutation Rates are Similar Within Pathways

**FIGURE 10:**
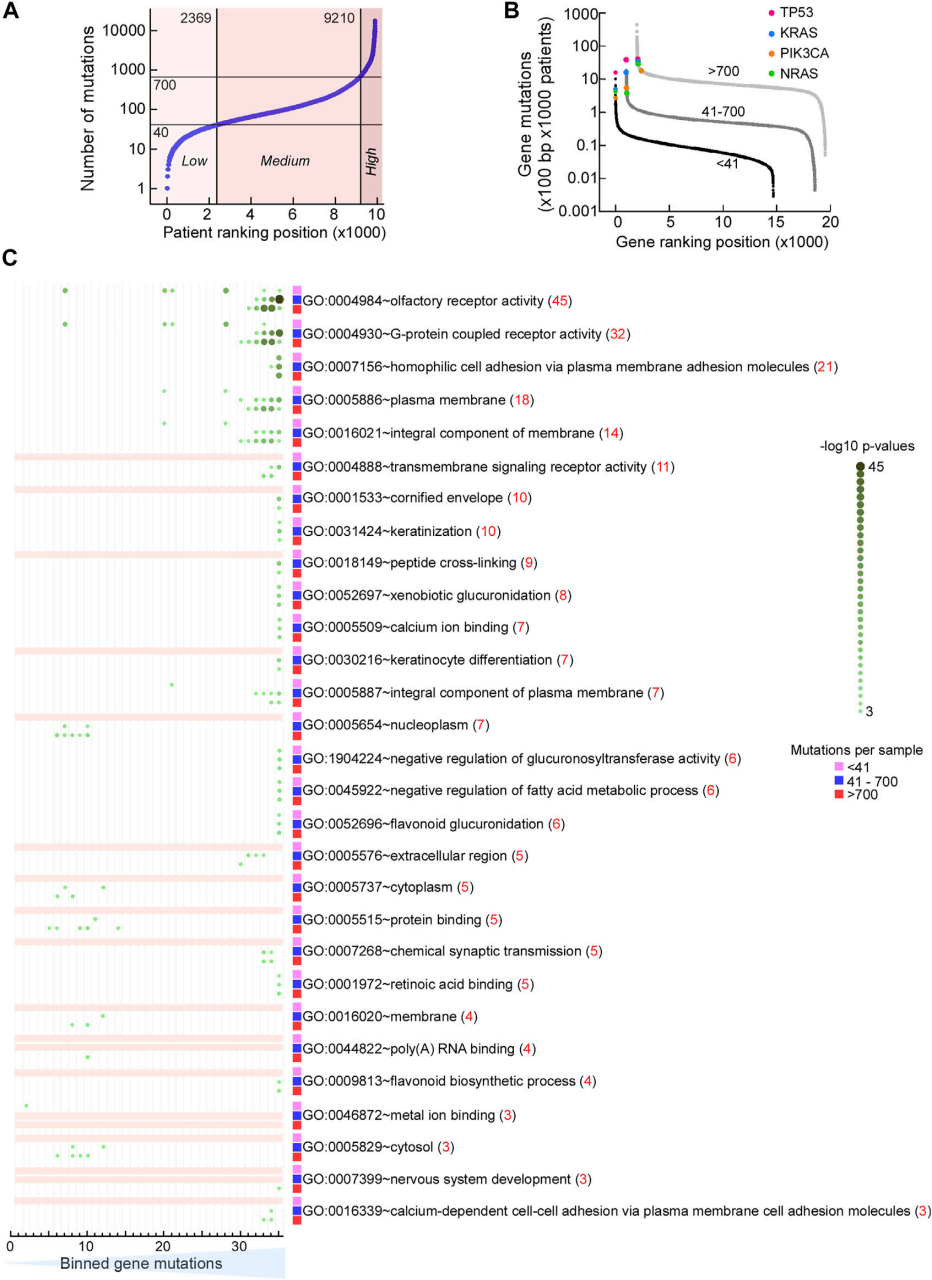
Mutation rates are similar within pathways. **(A)** Dot plot of TCGA samples ranked by the number of simple mutations in coding gene regions. *Horizontal reference lines,* cutoffs for number of mutations for “low,” “medium,” and “high” mutation load groups (MLGs); *vertical reference lines,* ranking position cutoffs. **(B)** Dot plot of ranked normalized numbers of coding gene mutations in the “low” (*black*), “medium” (*dark gray*), and “high” (*light gray*) MLGs. *Color*, ranking position of commonly mutated oncogenes. **(C)** Heat map of Gene Ontology (GO) terms for genes ranked in **(B)** for the three MLGs. Ranked genes in **(B)** were split into 35 bins of increasing mutational loads (*x*-axis) so that each bin contained ∼400 genes, irrespective of MLG. Each set of 400 genes was then used to conduct a GSEA using DAVID (https://david.ncifcrf.gov) and the strongest Benjamini–Hochberg-corrected *p*-value for each GO term within each MLG (*red value in parenthesis*) was then used to rank the results. For all three MLGs, the most mutated genes (bins ∼30–35) were the olfactory receptor genes (top 2 GO terms) and plasma membrane molecules involved in homophilic cell–cell adhesion (GO terms 3–5). *Color-coding and size scale*, Benjamini–Hochberg-corrected *p*-values within individual bins. *Pink rectangle*, no significant enrichment.

The genes we analyzed here are broadly classified by the term “DNA repair.” In cancer, mutation rates vary both with respect to tumor type as well as within the same type of tumor. Therefore, we thought that it would be informative to consider the mutability of our DNA repair genes with those genome-wide, particularly in light of the view that only a handful of mutations drive tumorigenesis with most other mutations being considered innocuous (passenger mutations). In general, mutations that inhibit a major DNA repair process may substantially increase genome instability, as seen for example by inhibition of MMR versus DNA damage (McMurray and Tainer, 2003). In the 9,891 patient samples from COSMIC that were part of The Cancer Genome Atlas (TCGA) cohort, we recorded a >4 orders-of-magnitude range in the number of mutations (Figure 10A). We divided arbitrarily this cohort into 3 mutational load groups (MLGs), the low group having up to 40 mutations per sample, the medium group having between 41 and 700 mutations per sample, and the high group with greater than 700 mutations per sample. For each gene in these three groups, we computed the number of mutations that occurred every 100 bp of coding sequence in 1,000 patients (Gene mutations) and ranked the genes according to gene mutations. This plot allows us to detect the genes that are most mutated in each group. Protein-coding genes displayed an ∼4 orders-of-magnitude range in normalized mutation (mutations × 100 bp × 1,000 patients) in each MLG, extending to a near 6 orders-of-magnitude range for the combined MLGs. Surprisingly, canonical tumor suppressors and oncogenes regarded as most frequently mutated were not among the top hits in the medium and high MLGs; in fact, in the high MLG TP53 ranked 65th, with a normalized mutation ∼4- to 10-fold lower than the top hits, where several protocadherin genes were included (Figure 10B). Thus, selection pressure towards tumorigenesis, which depends heavily on canonical tumor suppressors and oncogenes (e.g., TP53, KRAS, PIK3CA, NRAS, and several others) in patients with low mutation rates, may shift to a different and larger mutation set in patients with high mutation rates.

**FIGURE 11:**
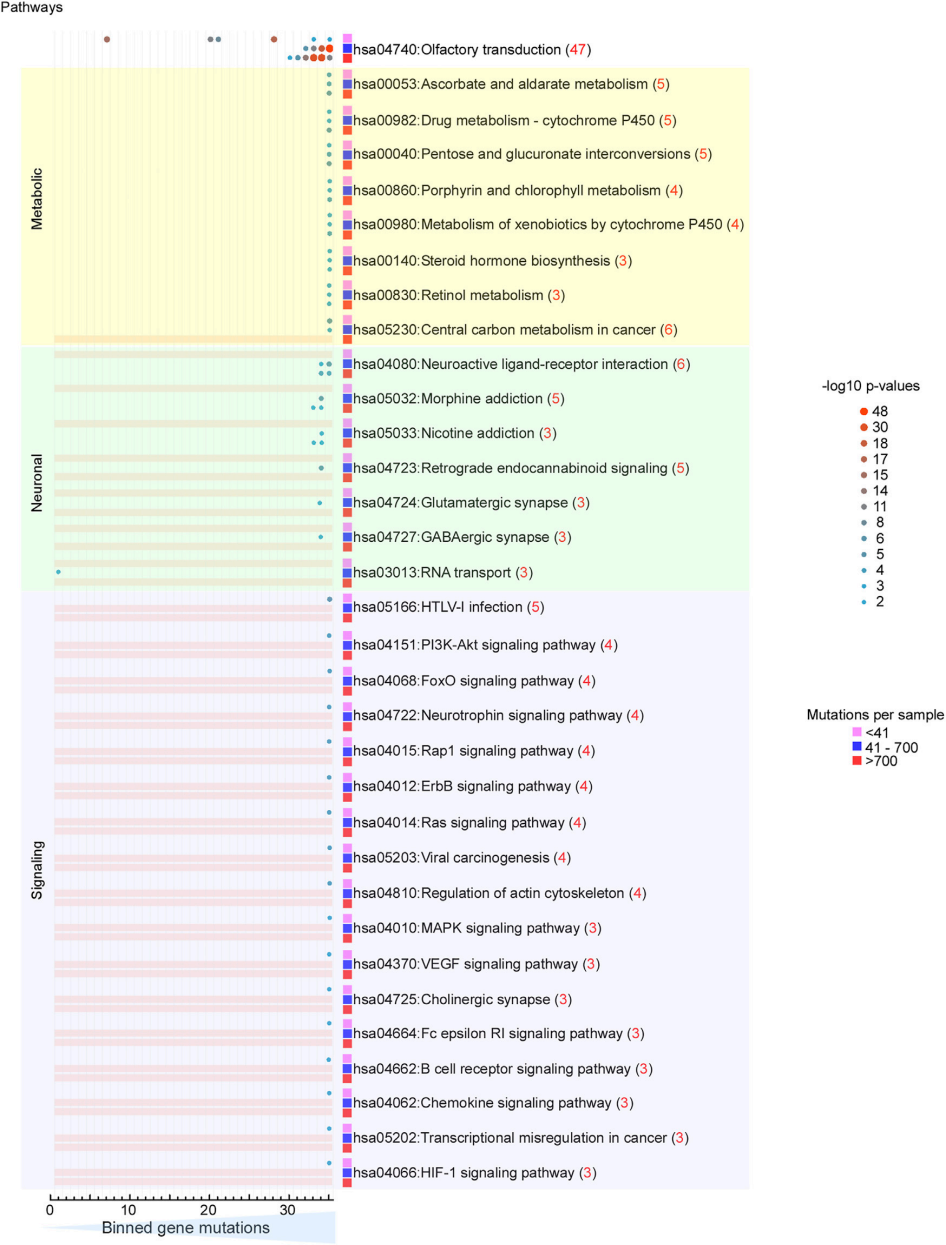
Signaling pathways are selectively mutated in patients with “low” mutation loads. Heat map of KEGG pathway analysis of GSEA. *Yellow background*, highly mutated metabolic genes observed in all three MLGs. *Pale green background*, highly mutated neuronal pathways only detected in the “medium” and “high” MLGs. *Light purple background*, highly mutated signaling pathways selectively mutated in the “low” MLG. *Color-coding and size scale*, Benjamini–Hochberg-corrected *p*-values within individual bins. *Pink rectangle*, no significant enrichment.

To further characterize the nature of differentially mutated genes, for each MLG, we divided the ranked mutated genes into 35 bins, each bin containing ∼400 genes, and conducted a gene set enrichment analysis (GSEA). Surprisingly, ∼30 significant terms were revealed according to the Gene Ontology (GO) (Figure 10C) annotation and the KEGG pathways (Figure 11), and 15 enriched terms were recorded by InterPro (Supplementary Figure S2A). The most striking finding was the high mutability of olfactory receptor genes in all 3 MLGs, with −log10 *p*-values exceeding 40, and that of cadherin/protocadherin genes involved in homophilic cell adhesion, with −log10 *p*-values >20. Importantly, KEGG distinguished the low from the medium and high MLGs based on the high mutation rates within signaling pathways known to lead to tumorigenesis (Figure 11). In contrast, genes belonging to several metabolic pathways incurred high mutation rates in all 3 MLGs, whereas high mutation rates in neuronal-associated genes distinguished the medium-high MLGs. In sum, our analysis supports the concepts that (1) mutation rates tend to be similar for genes belonging to the same pathway, and (2) signaling pathways are more likely mutated than other gene families in cancer patients with low mutation rates.

### DNA Repair Genes are Selectively Targeted for Mutation in MLG Medium and High

**FIGURE 12:**
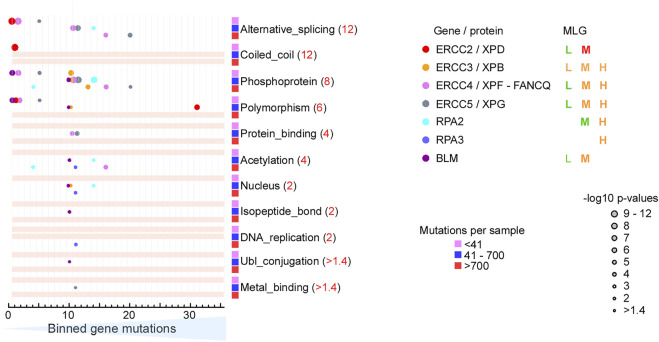
DNA repair genes incur low mutation rates in cancer genomes. Heat map of UniProtKB keywords for the DNA repair genes in this study from GSEA. *Color-coding and size scale*, Benjamini–Hochberg-corrected *p*-values for each individual DNA repair gene within each bin. Occasionally 2–3 genes fell within the same bin. *Pink rectangle*, no significant enrichment. MLG color coding: *green*, mutated at low rates (bins 1–5); *orange*, mutated at medium rates (bins 10–20); *red*, mutated at elevated rates (bin 31).

The MLG and GSEA classifications provided the backdrop against which we analyzed the mutation rates for our DNA repair genes. Seven of the nine genes occurred within significantly enriched GSEA terms following UniProtKB (Figure 12). Strong *p*-values were observed for the association with alternative splicing and post-translational phosphorylation in all 3 MLGs. Interestingly, the medium and high MLGs displayed strong association with acetylation, a post-translational modification that is acquiring prominence for enabling repair, by mediating the interaction of DNA repair factors with chromatin components (Bhakat et al., 2020; Bacolla et al., 2021). Overall, our DNA repair genes segregated with other genes with which they shared functional properties, such as “alternative splicing,” “phosphorylation,” and “acetylation.” These properties were increasingly targeted as the mutational load of patients increased from low to medium-high, suggesting selection toward malignant status in these two patient groups.

### Mutation Rates Decrease With Gene Age

**FIGURE 13:**
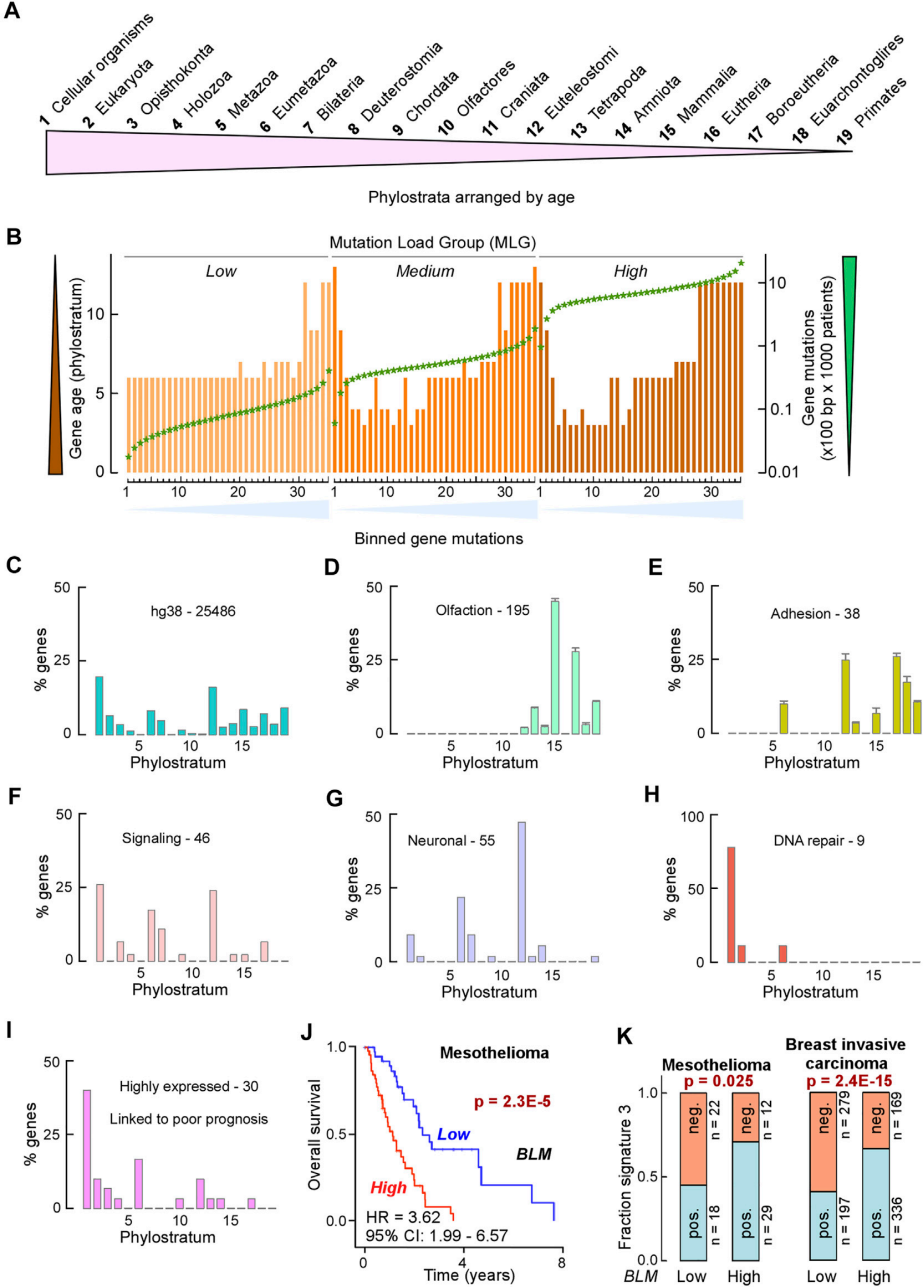
Mutation rates decrease with gene age. **(A)** Cartoon of phylostrata arranged by age from the origin of life to now. **(B)** Bar graph displaying the median age (as phylostratum, *left scale*) and the median of normalized mutations (×100 bp × 1000 cancer patient samples, *green stars*) for each of the 35 bins containing ranked mutated genes (see Figure 10). **(C–I)** Bar graph of percent genes in each of the 19 phylostrata. **(C)** Whole genome; **(D)** GO:0004984 (olfactory receptor activity) from Figure 10 (mean ± SE from low, medium and high MLGs); **(E)** GO:0007156 (homophilic cell adhesion *via* plasma membrane molecules) from Figure 10 (mean ± SE from low, medium and high MLGs); **(F)** combined signaling pathways for the low MLG from Figure 11; **(G)** combined neuronal KEGG terms for the medium MLG from Figure 11; **(H)** DNA repair genes in this study; **(I)** 30 genes most frequently associated with poor prognosis when overexpressed (Hambarde et al., 2021). **(J)** Kaplan–Meier curve of TCGA mesothelioma patients with low (below mean) and high (above mean) *BLM* mRNA levels. Hazard ratio (HR) and confidence interval (CI) are given. *p*-value from logrank test. **(K)** Bar plot of fraction of TCGA patients with low or high *BLM* mRNA levels exhibiting patterns of single-base substitutions conforming to signature 3 positive (pos.) or negative (neg.) in mesothelioma (*left*) and breast invasive carcinoma (*right*). *n*, number of samples; *p*-values from Fisher’s exact tests.

Cancer is hypothesized to involve reversion from the multicellular back to a primordial unicellular state, such that “younger genes should be enriched in destabilizing mutations in cancer” (Bussey and Davies, 2021). We tested this hypothesis by following the gene age stratification of all human genes according to phylostratigraphy (Figure 13A) (Domazet-Loso and Tautz, 2010; Litman and Stein, 2019) and then assessing the median phylostratum for each of the 105 bins described above for the three MLGs (Figure 13B), along with the median for the normalized mutations (mutations ×100 bp × 1000 patients). Although the transition from one MLG to the next carried some overlap (first two bins of medium and high MLGs) possibly due to our arbitrary cutoff, gene age decreased as the number of mutations increased, for all three MLGs. This trend was particularly striking for the medium and high MLG, in which 3 and 8 bins, respectively, displayed a median phylostratum tracing back to organisms with uniflagellate cells (phylostratum 3) at low and intermediate mutations, but much younger genes at high mutations, with a median at genes that arose in jawed fish and terrestrial vertebrates (phylostrata 12–13). When considering the contribution of individual phylostrata, genes that arose during phylostrata 15 (mammals), 17 (hoofed and pawed animals), and 19 (primates) were most highly mutated in all three MLGs (Supplementary Figure S2B).

For the olfactory receptor and cell adhesion gene families, which were enriched among the most highly mutated genes in the three MLGs, the percentage of young genes was up to 5-fold higher than expected, with no genes older than phylostratum 5 (Figures 13C–E). By contrast, in cell signaling and neuronal pathways, which were heavily targeted for mutation in the low and medium-high MLGs, respectively, young genes were poorly represented, and the spike in phylostrata 15–19 was absent (Figures 13F,G; Supplementary Figure S2B).

Apart from *RPA3* (phylostratum 6) and *EXO5* (phylostratum 2), all other DNA repair genes in this study arose during the origin of life (phylostratum 1) (Figure 13H). Genome-wide, the percentage of mutated phylostratum 1 genes in the three MLGs was similar, although it was greater in the less mutated (bins 1–17) than in the more mutated genes (bins 18–35) (mean ± SD, 32.7 ± 4.3 vs. 27.7 ± 4.4, *p* = 4.8 × 10^−8^, two-tailed *t*-test). Only *ERCC2 (XPD)* and *ERCC5 (XPG)* were found beyond bin 17 in the medium and high MLGs; in the low MLG all DNA repair genes were found within bins 1–10 (Figure 12). Therefore, given the consistently strong representation of phylostratum 1 genes across the entire mutational range, the low mutability of these DNA repair genes is remarkable. In sum, our analysis supports and extends a correlation between gene age and mutability in cancer, with young genes being more mutable, as proposed (Bussey and Davies, 2021).

### Cancer Genes Linked to Poor Prognosis are Overrepresented in Phylostratum 1

A seminal analysis of 7 solid tumor types from TCGA suggested that tumorigenesis entails a breakage in the homeostatic coordination of gene expression between old and young genes, such that selected old pathways are strongly upregulated whereas younger pathways are repressed (Trigos et al., 2017). Therefore, we analyzed the stratigraphic age of 30 genes that we found most consistently upregulated in all TCGA tumor types and most frequently associated with poor survival when expressed at levels above the mean in the respective tumor types (Hambarde et al., 2021). These 30 genes, which include *BLM*, were overwhelmingly overrepresented (40%) among the pool of genes that made the origin of life possible (Figure 13I). *BLM* overexpression, in particular, was strongly associated with poor survival in mesothelioma (Figure 13J); these patients exhibited a genome-wide single-base substitution landscape consistent with deficient HDR (“signature 3” positive), as opposed to patients with low *BLM* expression, who manifested proficient HDR, as assessed by their “signature 3” negative status (Figure 13K; Supplementary Table S14) (Alexandrov et al., 2020). High- and low-expressing *BLM* tumors displayed a similar HDR-deficient and -proficient distinction in breast cancer (Figure 13K; Supplementary Table S14), where signature 3 has been most widely reported (Alexandrov et al., 2020). In addition, high *BLM*-expressing breast tumors were positive for signature 13 (Supplementary Table S14 **)**, which has been attributed to APOBEC cytidine deaminase activity. In sum, our results support and extend the notion that upregulation of these 30, and other, ancient genes is part of a concerted process in cancer that favors breaking the constraints of a multicellular control of cell division and reversion to a primordial unicellular and proliferation-uncontrolled status, facilitated by distinct DNA repair pathways.

### EA Scores Predict That ERCC2 (XPD) is Selectively Targeted for Mutation in Urinary Tract Carcinomas

**FIGURE 14:**
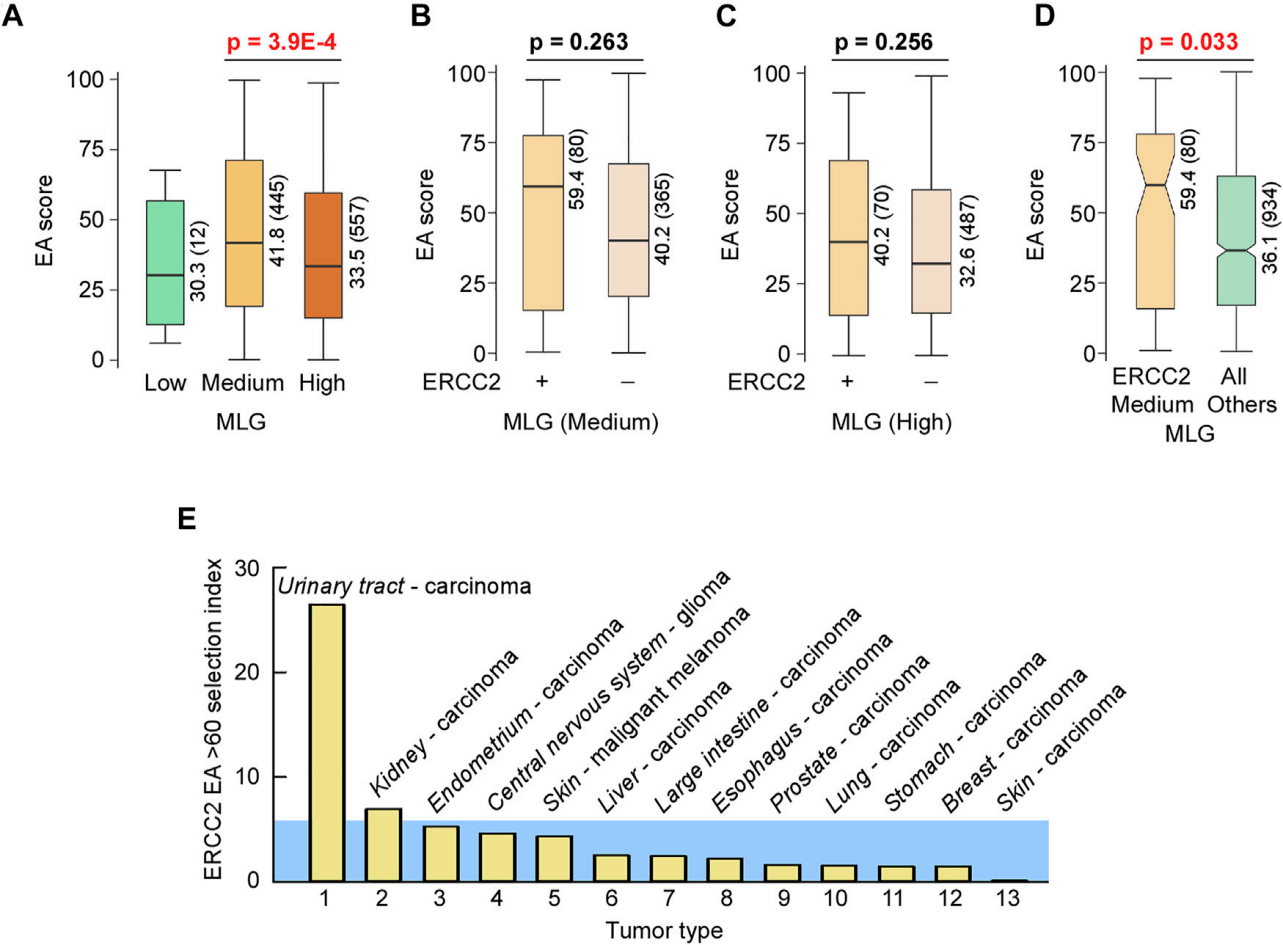
EA scores predict that *ERCC2* is selectively targeted for mutation in urinary tract carcinomas. **(A–D)** Box plots of EA scores for 1014 TCGA somatic variants found in *ERCC2*|*3*|*4*|*5*, *RPA1*|*2*|*3*, *BLM,* and *EXO5*: (**A**) the variants were divided into 3 Mutation Load Groups (MLGs); **(B)** the variants of the medium MLG were divided into *ERCC2* mutations (+) and mutations in the rest of genes (−); **(C)** the variants of the high MLG patients were divided into *ERCC2* mutations (+) and mutations in the rest of genes (−); **(D)** the *ERCC2* mutations of the medium MLG were compared to all other variants. **(E)** Strength of selection (selection index) for *ERCC2* mutations with EA scores ≥60 in various tumor types. *Blue highlight*, upper limit for the 99% confidence interval for all 13 selection index values.

Having computed EA scores based on conserved protein structure and obtained mutational maps, we assessed whether EA scores differed among the 3 MLGs. Of the total 1,014 missense substitutions in *ERCC2/3/4/5, RPA1/2/3, BLM*, and *EXO5* in TCGA, only 12 were found in the low MLG (Figure 14A), which, together with the mutational mapping data, indicate that these genes are not targeted for mutagenesis in patients with low mutational burden. EA scores were significantly higher in the medium than in the high MLG (Figure 14A); given that *ERCC2* was highly mutated in the medium MLG (bin 32, Figure 12), we asked whether such high EA scores would infer selective targeting of *ERCC2* for mutations during tumorigenesis in patients with medium MLG. Therefore, we computed the EA scores separately for patients with and without *ERCC2* mutations, for both the medium and high MLGs. The median EA score stood the highest in the medium MLG (Figures 14B,C), and indeed it was significantly higher than that for all other patients combined (Figure 14D). To further assess if *ERCC2* mutations might have been under selective pressure in any specific tissue, we computed the selection index for 13 tumors in which at least 3 mutated samples occurred. The selection index was clearly above the expected range in carcinomas of the urinary tract, in which 41 samples out of 763 harbored damaging *ERCC2* mutations (Figure 14E). We interpret these data to mean that damaging mutations in *ERCC2* (XPD) contribute to conferring tumor advantage in patients with medium levels of mutational burden, particularly in those affected by carcinomas of the bladder, renal pelvis, and ureter. In fact, *ERCC2* mutations could serve as a predictive biomarker for driving cisplatin responses that have been tested and validated in bladder cancers (Van Allen et al., 2014; Liu et al., 2016; Li et al., 2019).

## Discussion

Cancer development results from somatic mutation and clonal selection. Tumors carry an average of four coding substitutions under positive selection (Martincorena et al., 2017). A remarkably low negative selection on cancer point mutations means that identified coding mutations may unveil synthetic vulnerabilities for precision medicine. Fortunately, dramatic advances in sequencing identify single-site genetic and somatic disease mutations with potential to provide powerful insights into the functional biology of multi-functional helicase–nuclease complexes for precision medicine. Yet, gaining such insights requires efficient means to build molecular-based knowledge of mutant impacts in multiple different pathways and both enzymatic and structural roles. For example, whereas the related XPG and FEN1 nucleases both remove DNA secondary structures, their functions also include ability to fulfill structural roles and to avoid harmful interactions, such as template switching during DNA replication by sculpting DNA (Zheng et al., 2005; Tsutakawa et al., 2017; Tsutakawa et al., 2020a). We reasoned that as structure-specific nucleases and their regulating partners have key functions in replication and transcription, they may enable tumor survival and provide paths for the development of new therapeutic and prognostic tools, if their catalytic and structural roles can be better defined (Dehe and Gaillard, 2017). Indeed, structure-specific nucleases FEN1, XPG, and MRE11 have key structural, DNA sculpting, and catalytic roles that are controlled in different complexes (Shibata et al., 2014; Tsutakawa et al., 2017; Tsutakawa et al., 2020a). An example of the significance of pathway choice in biological outcome, XRCC1 links MRE11 and PolQ helicase to promote error-prone alternative end joining of DNA breaks (Eckelmann et al., 2020), but MRE11 initiation of homology-directed repair (HDR) at breaks is promoted by GRB2 adaptor (Ye et al., 2021).

In practice, biochemical and cell assays present different challenges, such as limited to a specific enzymatic or structural activity, poor feasibility for regular clinical practice, the use of commercial cell lines that may not recapitulate patient biology, plus cost and time requirements. Thus, an efficient and objective computational assessment of VUS that provides predictive reclassification may focus experimental verification to predicted pathological defects, such as defects in protein stability, DNA or partner protein binding, and hydrolytic activities. Furthermore, the results from benchmarked EA analysis (Katsonis and Lichtarge, 2017, 2019) may in some cases prove sufficient to guide clinical tests and decisions even in the absence of experimental data. These ET/EA analyses combined with structural mobility, as seen here for EXO5 (Hambarde et al., 2021), can reveal functional movements that could be blocked by inhibitors, as in the door stopper inhibitor used for the base repair enzyme uracil DNA glycosylase (UDG) that acts during replication (Nguyen et al., 2021).

Combining ET, disease, and EA80 with structure uncovers VUS likely to have meaningful defects by informing protein interactions, functional conformations, ATPase regulation, DNA interactions, and helicase and nuclease mechanisms. Yet, interpreting functional impacts for cancer and genome instability needs to include consideration of regulation and negative design. For example, mutation of a conserved Arg site of the abasic site nuclease APE1 makes a more efficient enzyme in terms of Kcat/Km (Mol et al., 2000). This Arg conservation slows the off-rate of the cleaved DNA product to enable a baton-passing handoff that avoids release of toxic and mutagenic intermediates (Wilson and Kunkel, 2000). Thus, evolutionary selection includes pathway regulation and avoidance of damaging events, and we can expect to see this in our ET/EA results on helicase–nuclease–RPA complexes. Specifically, tight DNA binding, nuclease activity, helicase activity, and functional flexibility can regulate and even change DNA repair pathways (Tubbs et al., 2009; Shibata et al., 2014; Yan et al., 2019; Hammel and Tainer, 2021; Whelan and Rothenberg, 2021). Mutations impacting the protein environment for metal ions including FeS clusters and other co-factors can also impact function, binding, and structure (Sundheim et al., 2006; Fan et al., 2008; Fuss et al., 2015). So, mutational defects in any of these properties can make an enzyme and pathway more or less efficient in repair at the cost of genome integrity or other negative consequences of reduced regulation.

Importantly, variant evaluation using ET/EA analyses in a complex or pathway manner may provide additional insights than analyzing an individual protein alone. For example, more EA80 VUS cluster in the DNA2 helicase domain than its nuclease domain, implying that its replication fork recovery role may be targeted by cancers rather than its DNA-end resection function. EA80 VUS on RPA is located on its OB-fold DBDs and away from the DNA binding path, suggesting key impacts onto RPA’s dynamic functions. In contrast, none of EA80 VUS targeted phosphorylation sites of RPA (N-terminal of RPA2) (Marechal and Zou, 2015), critical for DNA damage response and indicator of cancer progression: this implies that phosphorylation regulation is critical for cancer development and viability (Rector et al., 2017). To put our analyses in a context of protein complex, RPA-coated ssDNA is potentially lethal without timely displacement as it stimulates DNA damage response (Zou and Elledge, 2003). DNA2’s unique helicase function can displace RPA-coated ssDNA that other nucleases cannot (Zhou et al., 2015). Therefore, our ET/EA analyses on VUS reveal RPA functional interactions and a critical role of DNA2 helicase activity that act synergistically not only for genome stability but evidently also for tumor viability.

Most genomic alterations in tumors, including VUS, are regarded as passenger mutations with no known impact on tumor growth and progression. Our EA analysis coupled with a detailed mutational map enables the prediction that selection pressure is operative for at least some DNA repair genes, such as *ERCC2*, in patients with a medium mutational burden, particularly those affected by tumors of the urinary tract. Therefore, our composite analysis, showing a high degree of “concerted mutagenesis,” suggests a model whereby the binary distinction between “driver” and “passenger” mutations is replaced by a gradient scale, in which impact on tumorigenesis takes place both at a single gene level and at pathway and functional levels to enable tumor selection. Such a model is supported by recent data revealing an unexpected functional synergism of polygenic Fanconi anemia mutations (Tomaszowski et al., 2020).

Given the observed gradient scale, we tested using EA thresholds expected to have phenotypic impacts. From bacterial systems, stress responses to the master reactive oxygen defense enzyme superoxide dismutase (SOD) show a survival defect when SOD activity is reduced by 80% and detectable losses of [4Fe-4S] dehydratases (which superoxide reversibly inactivates) at ∼30% reduced SOD activity (Gort and Imlay, 1998). Guided by these quantitative observations on a key stress response enzyme, we chose EA values of 70–100 for predicted severe impacts and 30–70 for detectable impacts that may rise to levels that enable tumor selection at pathway and functional levels. We find that these EA thresholds capture evolutionarily important relationships. Yet, EA scoring may miss residues acting in sophisticated allosteric or side chain–main chain relationships, as we saw in our XPF and DNA2 analyses of EA-low scoring disease mutations. Systematic variant evaluation will therefore require combined structural and bioinformatic analysis as presented here, followed by further functional annotation and clinical interpretation.

Complementing our EA/ET site mapping, we employed a genome-wide mutability map that showed that mutations neither are random nor typically target individual genes. Rather, this map implies a selection path in cancer that draws advantage from targeting groups of genes belonging to specific pathways or sharing common functional processes as shown in our prototypic helicase–nuclease–RPA examples. Strikingly, the level of mutational “aggressiveness” is correlated within each pathway and process, but it varies by orders of magnitude with respect to which pathways are targeted and in which mutational groups these pathways operate. The most notable observation was the selective targeting of most, if not all, signaling pathways known to be relevant to cancer in patients with a low mutational burden. By contrast, neuronal-specific pathways were specifically targeted in patients with medium-high mutational loads. In a similar vein, processes such as alternative splicing, phosphorylation, and acetylation, which contained the DNA repair genes of this study, displayed a profound increase in instability as patient mutational burden increased from low to medium-high. These results suggest that there are multiple routes to cancer, and one comes from the combined negative impact of multiple genes being defective, which if occurred alone may not lead to cancer.

Our mutability map represents “the other side of the coin,” linking gene expression levels to mutation rates. For example, chromatin occupancy of BER complexes containing the DNA glycosylase NEIL1 is high in actively transcribed genomic regions but low in poorly transcribed genomic regions, thereby correlating high rates of oxidized DNA repair with low mutation rates, and *vice versa* (Bacolla et al., 2021). Therefore, tumors may target specific pathways and functions for mutation by coordinately tuning their gene expression levels. Mutability was also exquisitely dependent on gene age with mammal-specific genes that arose recently (past ∼65 million years), such as the large olfactory receptor gene family incurring the highest mutational rates in all three MLGs. Homophilic cell–cell adhesion with members that arose early in eumetazoa (867–604 million years ago) (Peterson and Butterfield, 2005) was also among the most targeted pathways in the 3 MLGs. Transcription of ancient genes is overall upregulated in the tumor relative to normal tissue found in the collection of 7 solid tumors, which comprises a subset of patients examined here (Trigos et al., 2017), whereas that of young genes is repressed, implying that tumorigenesis entails a breakage in the homeostatic control of gene expression between core cellular processes that arose early in single-celled organisms and more recent functions associated with multicellular organisms. Interestingly, XPB and XPD, but not other TFIIH proteins, are evolutionary retained in archaea, not in bacteria (Kelman and White, 2005). Perhaps the disruption of XPD regulation by TFIIH seen in somatic mutations plus its ability to function outside of TFIIH support cancer mutations toward a unicellular state (Ito et al., 2010).

### Summary and Prospects for Advances

Identification of complex and unique biological features associated with carcinogenesis provide therapeutic opportunities (Tsimberidou et al., 2020). For example, PARylation by PARP1 and dePARylation by PARG control recruitment and timing of repair events, so in tumors, the clinical success of PARP inhibitors (PARPi) can depend upon trapping PARP1 on the damaged DNA (Houl et al., 2019; Zandarashvili et al., 2020; Brosey et al., 2021). Breast tumors deficient in HDR show genomic alterations that can predict response to treatment with PARPi and other therapies that target DNA repair (Heeke et al., 2020). Genome instability and PARPi sensitivity can be caused by DNA repair defects, replication and transcription stress induced by genotoxic agents, dysregulated gene expression, and high frequency of mutations (Negrini et al., 2010; Gaillard et al., 2015; Rose et al., 2020). Thus, there are opportunities to induce PARPi sensitivities, for example, by administering alkylating agents to induce DNA single-strand breaks, to stall DNA replication forks, and to promote damage requiring efficient repair for transcription (Tubbs et al., 2007; Tubbs et al., 2009; Ying et al., 2012; Rose et al., 2020). As helicase–nuclease–RPA complexes can enable transcription and replication stress in cancer cells with impacts on tumor survival and genome instability, they may provide drug targets with the potential to be less pleiotropic than current kinase targets in the clinic. Toward this goal, our combined approach both provided insights into VUS and harnessed cancer mutation sites to decipher the challengingly complex biology of multi-functional helicase–nuclease–RPA complexes.

Viewed through the lens of EA, cancer mutations suggest how complexes integrate catalytic activity and structural roles by providing a picture of functional regions akin to saturation mutagenesis. Overall, we find that disease mutations are predicted to have severe impacts by EA scoring and primarily localize to structured domains, active sites, and interfaces. Is this because most EA80 VUS destabilize structures that cannot be done for unstructured regions? Due to its essential role in transcription, TFIIH is exceptional with only two disease mutations in XPB that map to non-helicase domains. In contrast, there are many XPB high EA VUS that mostly map to the helicase domains. Typically, mutations in both gene copies are requisite for autosomal recessive diseases to have a detectable phenotype. However, protein destabilization may be dominant negative in an assembly, as originally found for disease-causing point mutations in superoxide dismutase, a master regulator of reactive oxygen stress (Deng et al., 1993; Phillips et al., 1995; DiDonato et al., 2003; Shin et al., 2009; Pratt et al., 2014). Furthermore, we find evidence in XPD, RPA, and XPG of high EA VUS mutations that likely reduce regulatory interactions in higher eukaryotes that could similarly be dominant negative by relaxing regulatory control.

The effects of predicted high impact mutations for interfaces and assembly can be efficiently tested experimentally by high-throughput methods such as small angle x-ray scattering (SAXS), which includes data on flexible and intrinsically unstructured regions (Hura et al., 2013; Rambo and Tainer, 2013; Brosey and Tainer, 2019), and combined with x-ray crystallography and cryo-electron microscopy (Brosey et al., 2016; Horst et al., 2019). Furthermore, we can expect that our structurally informed ET/EA approach will strengthen with time as it benefits from the amount of mutational and structural data, which are dramatically increasing. Ultimately, we need integrated data to span from molecules to cells and humans (Brosey et al., 2017). Ongoing clinical trials combining HDR-targeted agents (such as PARPi) and immunotherapy could be enriched by comprehensive molecular profiling if NGS variant information can have prognostic impacts. New therapies increasingly target molecular mechanisms and are guided by the tumor mutational landscape. The integrated structural and EA approach reported here may enable combined analyses of multiple variants or polymorphisms within DNA repair pathways to provide an alternative way of analyzing their overall effects with relevance to genetic markers and therapeutic strategies. Robust objective structural and computation approaches to helicase–nuclease–RPA complexes could point to new paths for anti-cancer therapeutic strategies.

## Data Availability

The datasets presented in this study can be found in online repositories. The names of the repository/repositories and accession number(s) can be found in the article/Supplementary Material.
