# Supplementary material for: Decoding Cancer Variants of Unknown Significance for Helicase–Nuclease–RPA Complexes Orchestrating DNA Repair During Transcription and Replication
Source: Front Mol Biosci. 2021 Dec 14;8:791792. doi: 10.3389/fmolb.2021.791792 (PMC8710748; doi:10.3389/fmolb.2021.791792)
Supplement: Supplementary file 4 [file DataSheet1.PDF]

Supplementary Material

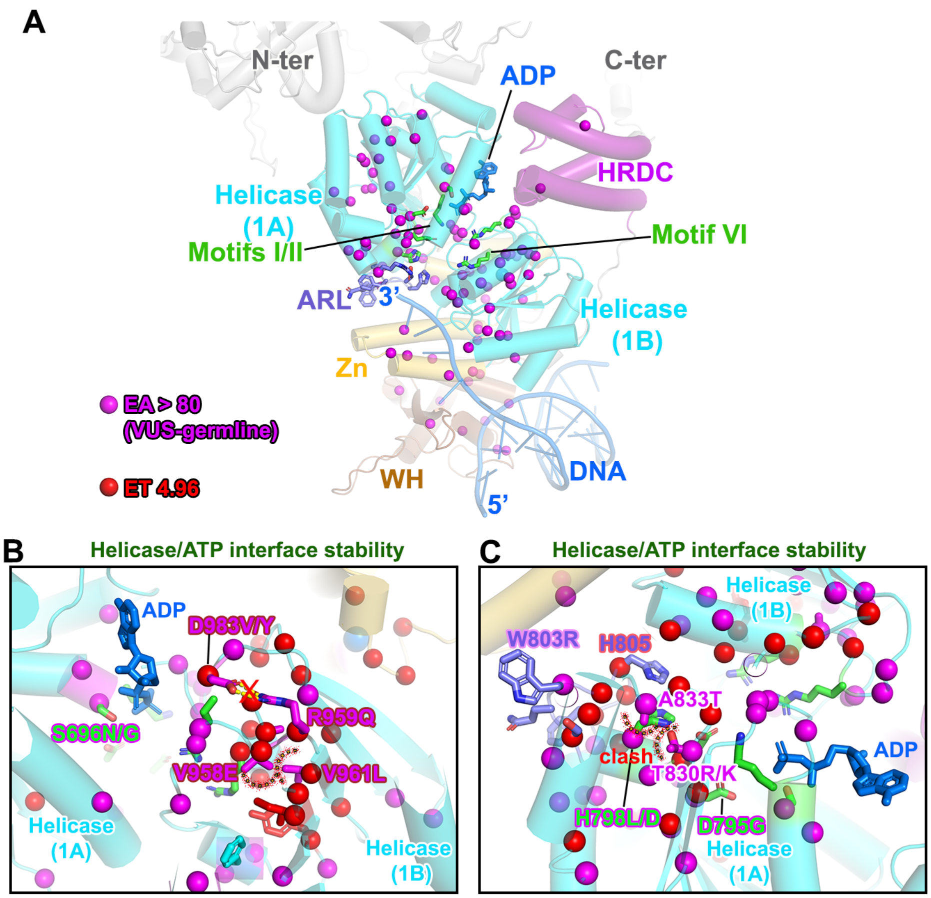

Supplementary Figure S1. Germline VUS with EA80 impacts helicase/ATP binding interface of BLM. **(A)** EA80 germline VUS are mapped on BLM structure. Colors correspond to Figure 6A. **(B–C)** Impacts of EA80 germline VUS on helicase/ATP interface of BLM.

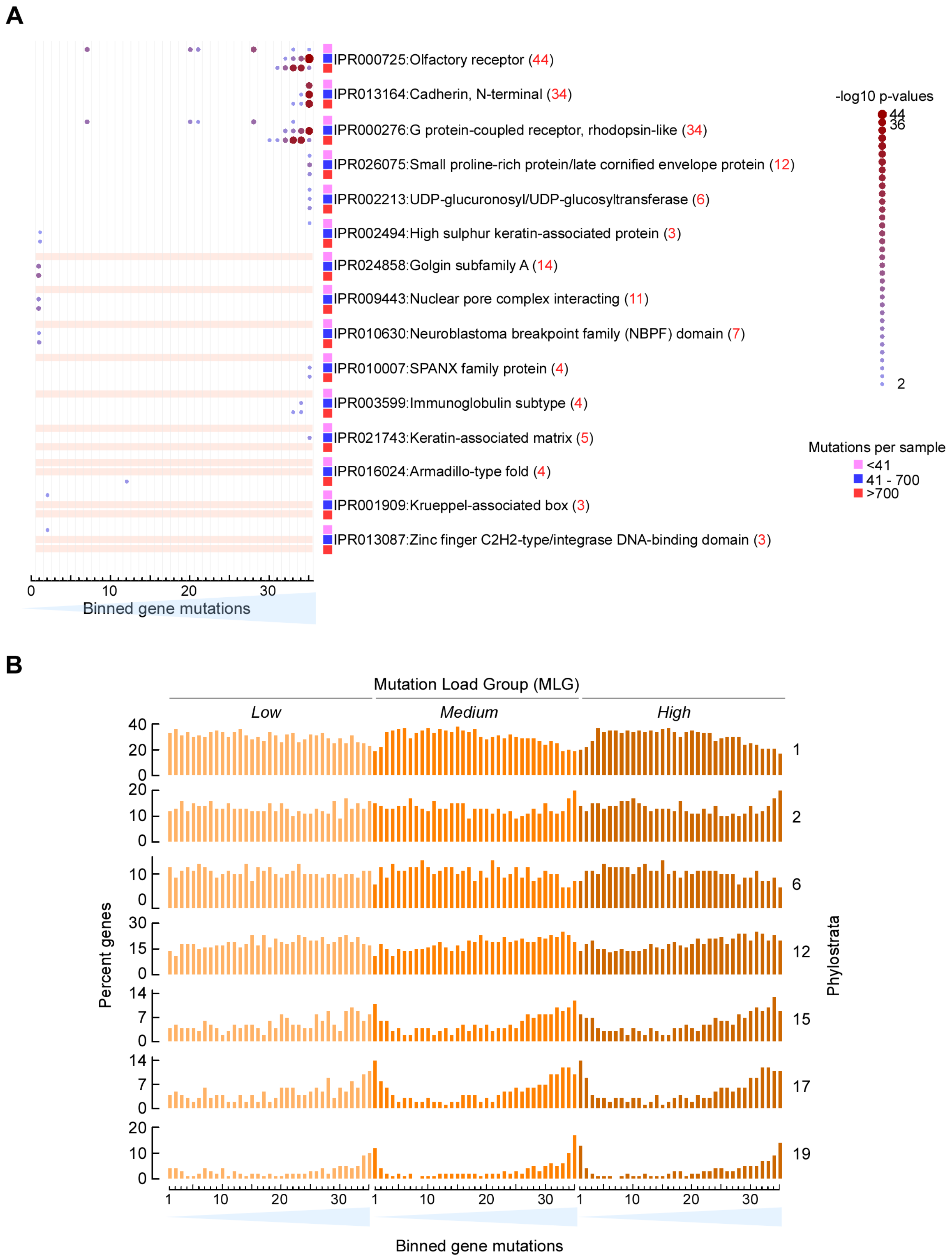

Supplementary Figure S2. Mutational rates from InterPro and gene mutations in different phylostrata. **(A)** Heat map of InterPro GSEA from the data in Figure 10. *Color-coding and size scale*,

Benjamini-corrected p-values within individual bins. *Pink rectangle*, no significant enrichment. **(B)** Bar graph displaying the percent genes within a given phylostratum in each of the 35 bins containing ranked mutated genes for the MLGs (see Figures 10 and 13). Only phylostrata reaching at least 10% genes are shown.

SUPPLEMENTARY TABLES

Supplementary Table S1. Data for Figure 1A. Patient risk and *ERCC5* mutations.

Supplementary Table S2. Data for Figure 1B. Patient risk and *ERCC5* expression

Supplementary Table S3. Data for Figure 1B. Patient risk and *XPA* or *XPC* expression

Supplementary Table S4. Data for Figure 1C. Kaplan Meier analysis for all available genes in LGG

Supplementary Table S5. Data for Figure 2A top. *ERCC5* gene expression in tumors and matched normal tissues from TCGA

Supplementary Table S6. Data for Figure 2A bottom. Location of genes whose expression was most highly correlated with that of *ERCC5* in tumors.

Supplementary Table S7. Data for Figure 2B. Genes whose expression was most highly correlated with that of *ERCC5* in tumors.

Supplementary Table S8. Data for Figure 2C. Genes co-expressed with *ERCC5* in tumors

Supplementary Table S9. Data for Figure 2D. Tumor types with simple mutations correlated to *ERCC5* expression.

Supplementary Table S10. Data for Figure 2E. Mutation signature of tumors identified by ERCC5 expression.

Supplementary Table S11. ET table for ERCC2-3-4-5, BLM, EXO5, DNA2, and RPA.

Supplementary Table S12. EA table for ERCC2-3-4-5, BLM, EXO5, DNA2, and RPA.

Supplementary Table S13. EA table for disease mutations in ERCC2-3-4-5, BLM, EXO5, DNA2, and RPA.

Supplementary Table S14. Top 30 gene age and *BLM* mutational signatures

SUPPLEMENTARY VIDEOS

Supplementary Video S1 *Related to Figure 3*. ERCC5 (XPG)

Supplementary Video S2 *Related to Figure 4*. ERCC4 (XPF)

Supplementary Video S3 *Related to Figure 5*. ERCC2 (XPD) and ERCC3 (XPB)

Supplementary Video S4 *Related to Figure 6*. BLM

Supplementary Video S5 *Related to Figure 7*. EXO5

Supplementary Video S6 *Related to Figure 8*. DNA2

Supplementary Video S7 *Related to Figure 9*. RPA
